# The protein family TcTASV-C is a novel *Trypanosoma cruzi* virulence factor secreted in extracellular vesicles by trypomastigotes and highly expressed in bloodstream forms

**DOI:** 10.1371/journal.pntd.0006475

**Published:** 2018-05-04

**Authors:** Lucas D. Caeiro, Catalina D. Alba-Soto, Mariana Rizzi, María Elisa Solana, Giselle Rodriguez, Agustina M. Chidichimo, Matías E. Rodriguez, Daniel O. Sánchez, Gabriela V. Levy, Valeria Tekiel

**Affiliations:** 1 Instituto de Investigaciones Biotecnológicas—Instituto Tecnológico de Chascomús (IIB-INTECH), Universidad Nacional de San Martín (UNSAM)—Consejo Nacional de Investigaciones Científicas y Técnicas (CONICET), San Martín, Buenos Aires, Argentina; 2 Departamento de Microbiología, Parasitología e Inmunología, Facultad de Medicina, Universidad de Buenos Aires, Instituto de Investigaciones en Microbiología y Parasitología Médicas (IMPaM), UBA-CONICET, Buenos Aires, Argentina; 3 Departamento de Cs. Básicas, Universidad Nacional de Luján, Luján, Buenos Aires, Argentina; Tulane University School of Public Health and Tropical Medicine, UNITED STATES

## Abstract

TcTASV-C is a protein family of about 15 members that is expressed only in the trypomastigote stage of *Trypanosoma cruzi*. We have previously shown that TcTASV-C is located at the parasite surface and secreted to the medium. Here we report that the expression of different TcTASV-C genes occurs simultaneously at the trypomastigote stage and while some secreted and parasite-associated products are found in both fractions, others are different. Secreted TcTASV-C are mainly shedded through trypomastigote extracellular vesicles, of which they are an abundant constituent, despite its scarce expression on culture-derived trypomastigotes. In contrast, TcTASV-C is highly expressed in bloodstream trypomastigotes; its upregulation in bloodstream parasites was observed in different *T*. *cruzi* strains and was specific for TcTASV-C, suggesting that some host-molecules trigger TcTASV-C expression. TcTASV-C is also strongly secreted by bloodstream parasites. A DNA prime—protein boost immunization scheme with TcTASV-C was only partially effective to control the infection in mice challenged with a highly virulent *T*. *cruzi* strain. Vaccination triggered a strong humoral response that delayed the appearance of bloodstream trypomastigotes at the early phase of the infection. Linear epitopes recognized by vaccinated mice were mapped within the TcTASV-C family motif, suggesting that blockade of secreted TcTASV-C impacts on the settlement of infection. Furthermore, although experimental and naturally *T*. *cruzi*-infected hosts did not react with antigens from extracellular vesicles, vaccinated and challenged mice recognized not only TcTASV-C but also other vesicle-antigens. We hypothesize that TcTASV-C is involved in the establishment of the initial *T*. *cruzi* infection in the mammalian host. Altogether, these results point towards TcTASV-C as a novel secreted virulence factor of *T*. *cruzi* trypomastigotes.

## Author summary

*Trypanosoma cruzi* is the kinetoplastid parasite that causes Chagas’ disease, a neglected infection endemic in Latin America and emerging worldwide. Being vaccines currently unavailable and treatments not completely effective, identification and characterization of parasite molecules that can be target for these interventions are urgently needed. Of particular interest are surface anchored and secreted proteins involved in parasite—host interplay. Recently, extracellular vesicles released from protozoan pathogens have been shown to alter host cell function favoring the establishment of infection. Trypomastigotes are the disseminating stage of *T*. *cruzi*, being their presence in peripheral blood a hallmark of early acute infection in mammals. While the most abundant proteins of the trypomastigote surface are fairly well characterized, little is known about other, less abundant and more recently discovered multigenic families, which could have critical functions in the parasite—host interaction. The *T*. *c**ruzi* Trypomastigote Alanine, Valine and Serine rich proteins (TcTASV) belong to a medium-size multigene family of ~40 members that remained unobserved until a few years ago when it was identified through a trypomastigote-enriched cDNA library. Almost simultaneously, an expression library immunization approach designed to discover novel vaccine antigens in *T*. *cruzi*, spotlighted the TcTASV-C subfamily, as a fragment of a TcTASV-C gene was identified in a pool of protective clones. A distinctive feature that characterizes TcTASV proteins–and particularly the TcTASV-C subfamily- is their predominant expression in trypomastigotes. Recent transcriptomic and proteomic studies uphold our previous observations that the TcTASV family is over-represented in the trypomastigote stage, and therefore could represent an interesting target for rational intervention against *T*. *cruzi* infection. Here show that TcTASV-C is mainly secreted through extracellular vesicles (EVs) of trypomastigotes, and is a major cargo of its content. We have also shown that TcTASV-C is much more expressed in trypomastigotes purified from blood from infected mice than in trypomastigotes harvested from *in vitro* cultures, suggesting that host molecules should trigger TcTASV-C expression *in vivo* during the infection. The immunization of mice with TcTASV-C interfered with the early acute phase of *T*. *cruzi* infection through a strong humoral immune response. TcTASV-C should be considered as a novel secreted virulence factor of *T*. *cruzi* trypomastigotes and -although its biological function is still unknown- we hypothesize its participation in the early steps of *T cruzi* infection in the mammalian host.

## Introduction

*Trypanosoma cruzi*, is the kinetoplastid pathogen that causes Chagas’ disease. There are about 10 million people currently infected and more than 50–60 million people living in endemic areas, at risk of infection. Chagas’ disease is a chronic debilitating illness with symptoms generally appearing 10 or more years after the initial infection [1–3]. At this stage, anti-parasitic drugs are poorly effective and patients are treated according to their cardiac, digestive or neurological compromise [4–5]. Acute infection is usually undetected because of its mild and unspecific symptoms and, therefore, the patients are not diagnosed. The acute phase of the infection is characterized by the presence of high levels of trypomastigotes in blood. These nonreplicative trypomastigotes invade nucleated cells, where they differentiate to the amastigote stage that replicates in the cytoplasm and differentiates again to trypomastigotes. Then, the infected cell bursts and trypomastigotes are released once again to circulation. During the acute phase this cycle repeats itself actively and the trypomastigote disseminates the infection to several organs and tissues [6]. Considering the absence of preventive or chemoprophylactic vaccines as well as the life cycle of the parasite, uncharacterized molecules differentially expressed in the infective trypomastigote stage can be interesting novel targets for rational intervention against Chagas’ disease [7,8].

The *T*. *c**ruzi* Trypomastigote Alanine, Valine and Serine (TcTASV) rich proteins belong to a medium-size multigene family of ~40 members that was identified from a library of trypomastigote-enriched mRNAs [9]. The TcTASV family is conserved among all the *T*. *cruzi* lineages analyzed so far and has no orthologues in other species, including the closely-related trypanosomatids *T*. *brucei*, *T*. *rangeli* and *Leishmania sp*. [9]. TcTASV proteins, whose function is still unknown, are expressed mainly in the trypomastigote stage. The N- and C-terminal regions of the TcTASV proteins possess a signal peptide and a consensus for a GPI anchor addition, respectively, and display the highest level of conservation, while the central region presents more variability [9]. TcTASV family can be distinguished by the common amino acid motif *tasv_all* that starts approximately at amino acid 42 (Vx_1_x_2_x_3_[CES]x_4_x_5_TDGx_6_Lx_7_Wx_8_x_9_x_10_x_11_Ex_12_x_13_Wx_14_x_15_Cx_16_x_17_x_18_P). The TcTASV family is comprised of 4 subfamilies -TcTASV-A, B, C and W- defined by the primary amino acid sequence and length of polypeptides. Further, each subfamily presents certain amino acids at the indeterminate positions (x_1_, x_2_, etc) of the *tasv_all* motif. For example, subfamilies TcTASV-C and TcTASV-A both have proline and glycine at positions X_4_ and X_5_, while TcTASV-B contains serine and arginine, and TcTASV-W has alanine at X_4_ and glutamic acid at X_5_.

The TcTASV-C subfamily includes approximately 15 genes (small variations are found in different strains) with protein products of 330–360 amino acids [9, 10]. A few years ago, in the search for novel vaccine candidates by a genetic immunization approach, a fragment of a TcTASV-C gene (TcCLB.511675.3; ID TritrypDB, [11]) was identified among a pool of antigens that protected mice from a parasite challenge with a highly virulent *T*. *cruzi* strain [12]. In a first characterization of the TcTASV-C subfamily we found that TcTASV-C is a thickly glycosylated ~60 kDa polypeptide, expressed in trypomastigotes and absent in all other parasite stages [10]. TcTASV-C presents a characteristic distribution pattern of scattered dots along the parasite surface and flagellum, and is spontaneously secreted to the medium. While anti-TcTASV-C antibodies are detected in about 30% of chronically-infected patients, the seroprevalence in reservoir dogs with active infection rises to 75% [10,13]. In the experimental murine *T*. *cruzi* model, TcTASV-C specific antibodies can be detected early from the beginning of the infection [10]. Although TcTASV-C proteins are not major components of the parasite in trypomastigotes derived from *in vitro* cultured cells [10], several TcTASV-C peptides have been recently identified in secretomes of *T*. *cruzi* trypomastigotes [14–16]. Interestingly, TcTASV peptides were found in the bloodstream trypomastigote proteome, but not in proteomes from *in vitro* cultured cell derived trypomastigotes [17]. Also, a recent analysis of an overall transcriptome of the host cell and *T*. *cruzi* during the course of infection confirmed that the TcTASV family is extensively over represented in trypomastigotes, relative to all the other stages of the parasite, and several TcTASVs mRNAs are among the most abundant in the trypomastigote stage [18]. After being unnoticed for several years, and in agreement with our previous reports, these findings also outpoint towards TcTASVs as potential virulence factors and as interesting targets for study and rational intervention.

Here we present results leading to a deeper understanding of the TcTASV-C subfamily and its performance as a vaccine antigen.

## Results

### TcTASV-C motif discovery

**Fig 1:**
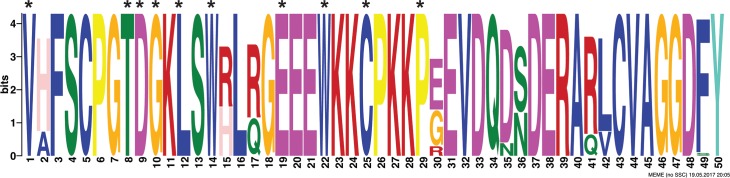
The *tasv_c* motif is distinctive for all TcTASV-C proteins. Graphical representations of patterns were generated after multiple sequence alignment of all TcTASV-C protein sequences from different strains (n = 39; S1 Dataset) with WebLogo [19]. The *tasv_c* motif starts at amino acids 42–43 of most TcTASV-C proteins and encompass 50 amino acids. Asterisks denote conserved amino acids of *tasv_all* motif.

First, a bioinformatic analysis was carried out to determine whether there was a common pattern among all TcTASV-C members. A distinctive and conserved *tasv_c* motif of 50 amino acids was identified in all TcTASV-C proteins (Fig 1). In most proteins, the *tasv_c* motif starts at amino acid 42–43, including and expanding the previously reported *tasv_all* motif, common for all TcTASV proteins irrespectively of their subfamily (asterisks in Fig 1). Only TcTASV-C proteins are retrieved by searching any database with the *tasv_c* motif.

### Several TcTASV-C genes are simultaneously expressed in the trypomastigote stage

**Fig 2:**
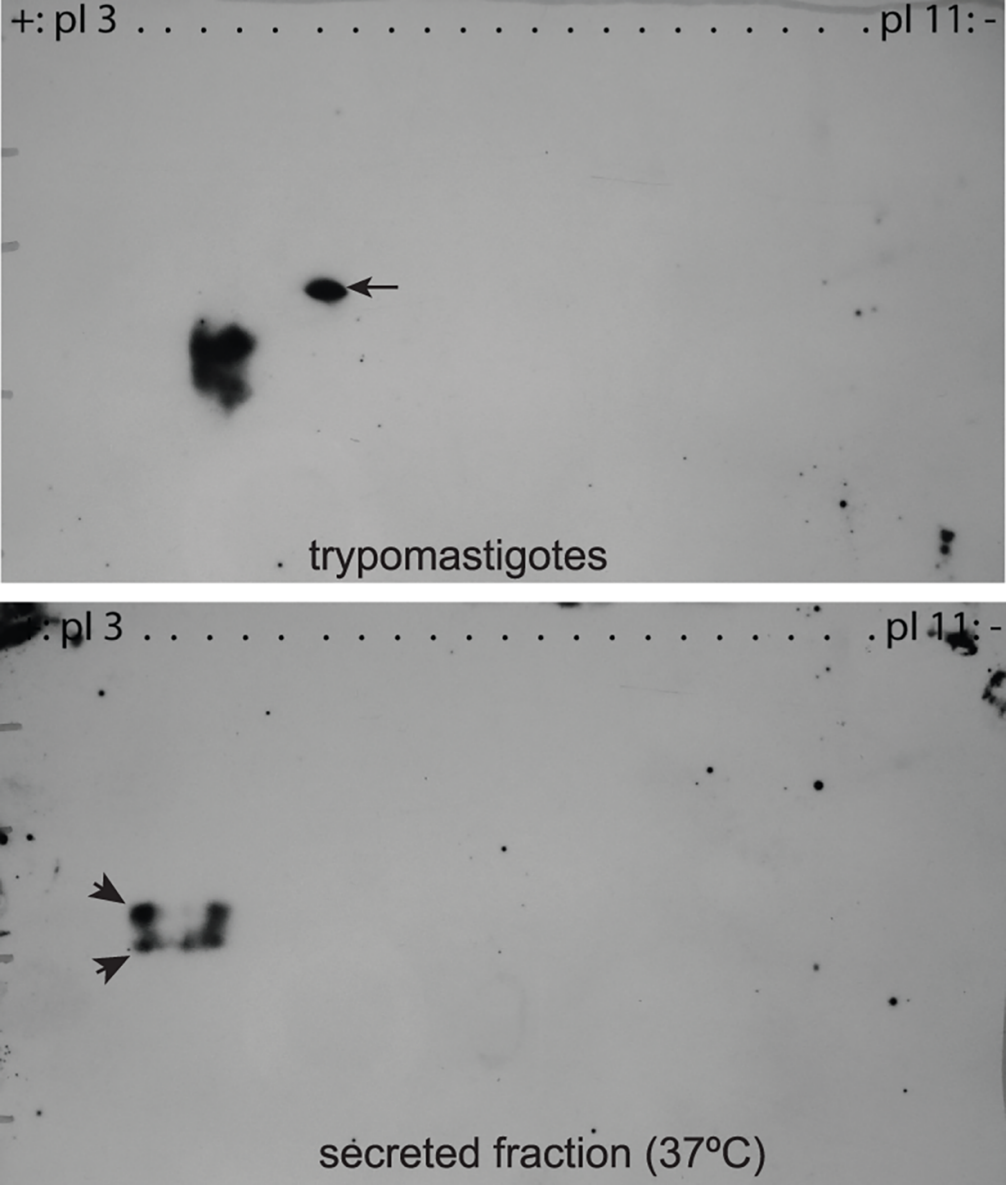
Several TcTASV-C genes are simultaneously expressed at the surface of trypomastigotes and secreted to the medium. CL Brener trypomastigotes were incubated for 2 h at 37°C in serum-free medium; trypomastigotes (pellet, upper panel) and secreted fraction (supernatant, bottom panel) were processed by 2D electrophoresis and analyzed by western blot.

We have already described that TcTASV-C is expressed both at the parasite surface and secreted to the medium [10]. To investigate whether–among the 15 TcTASV-C genes of CL Brener strain- several genes (or only one) are simultaneously expressed, and to clarify if surface-located and secreted TcTASV-C proteins are expressed from the same genes, we undertook a 2D gel based approach (Fig 2). More than one TcTASV-C product was observed both in the parasite-associated and in the secreted fractions (Fig 2), suggesting that more than one TcTASV-C gene are simultaneously expressed. On the other hand, there were common and differential TcTASV-C spots detected in both fractions. There was a clear band of higher molecular weight only present in the parasite fraction (arrow in Fig 2, upper panel) and two bands of more acidic isoelectric point (pI) in the secreted one (arrowheads in Fig 2, lower panel). Two other bands seemed to be shared by both samples.

These results show that the expression of different TcTASV-C genes occurs simultaneously at the trypomastigote stage and suggest that while some secreted and parasite-associated products are found in both fractions, others are different.

### Most of the TcTASV-C produced by CL Brener trypomastigotes is secreted through extracellular vesicles

TcTASV-C is secreted and also detected–by immunofluorescence microscopy- at trypomastigote surface in spots that are compatible with detergent resistant domains [10]. These domains are often associated with secretion of molecules through extracellular vesicles (EVs) [20,21]. In this context, we investigated whether TcTASV-C proteins were released associated with EVs or as soluble factors (VF: vesicle-free fraction). In a first set of experiments, trypomastigote- derived conditioned media was resolved by ultracentrifugation in density gradients; TcTASV-C was detected in fractions corresponding to extracellular vesicles (S1 Fig).

**Fig 3:**
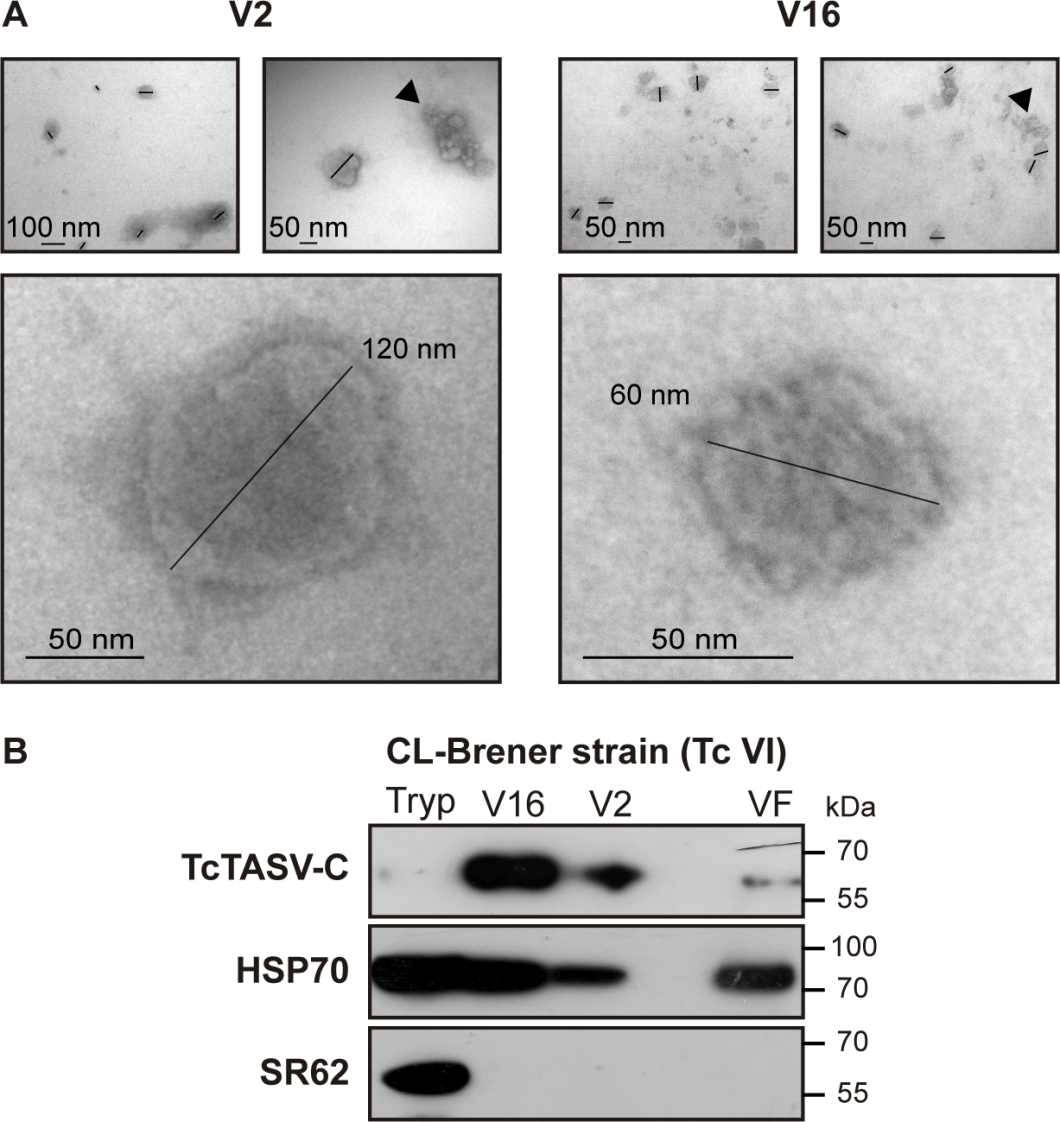
TcTASV-C is mostly secreted in EVs from trypomastigotes of CL Brener strain. **(A)** Representative TEMs of large (V2) and small (V16) EVs. Vesicles are indicated by black bars and clusters formed by ultracentrifugation are marked with black arrowheads. (**B)** 30x10^6^ trypomastigotes and the secretion equivalent of small EVs (V16), large EVs (V2) and EV-free fraction (VF) were processed by western blot with antisera against TcTASV-C, HSP70 and TcSR62.

We next investigated whether TcTASV-C proteins were released associated with large (V2) or small (V16) EVs, whose presence and purity was confirmed by transmission electron microscopy (TEM; Fig 3A). All samples showed vesicles of 30–130 nm in size after 2 h of ultracentrifugation, while vesicles obtained after 16 h were smaller (~50 nm; Fig 3A). TcTASV-C was secreted in both EVs populations in the CL Brener strain (Fig 3B); in the small EV fraction (V16, Fig 3B, upper panel) TcTASV-C appeared as a highly abundant component, while it was almost undetectable on parasite pellets at these conditions. This agrees with the already reported low level of expression of TcTASV-C on parasite body in cell derived trypomastigotes [10]. Longer exposure times were needed to evidence the TcTASV-C expression on trypomastigotes but render overexposed and unclear images for V2 and V16 fractions. As expected, the heat shock protein 70 (HSP70), a secretome marker, was detected in all fractions and, TcSR62, a nucleo-cytoplasmic and non-secreted RNA binding protein, only in the parasite pellet (Fig 3B) [10,22,23].

A proteomic analysis of V2 and V16 secreted extracellular vesicles of CL Brener strain was also carried out, to confirm our western blots results and, besides, because there are no proteomic analysis of V2 and V16 cargo proteins of trypomastigotes. Indeed, the data currently available from exoproteomes of trypomastigotes were derived from total secreted material or from a mixture of vesicles from parasites and host cells purified together [14–16]. High confidence peptides of four TcTASV-C genes were found both in V2 and V16 samples (TcCLB.508741.440, TcCLB.509123.10, TcCLB.509147.40, TcCLB.508737.10), which is in line with the 2D western blot results. Although TcTASV-C peptides were detected in both EV fractions, is noteworthy that each EV population presented a differential set of major proteins (V2: n = 271; V16: n = 189), and only a minor core of 142 common proteins (among which are the TcTASV-C peptides) (S2 Fig). This suggests that both fractions of vesicles correspond to different populations. Surface, intracellular as well as a considerable percentage of hypothetical proteins were identified by proteomics in trypomastigote EVs (S2 Fig). In context, the picture obtained could indicate that–at least at the assayed conditions- the paucity of TcTASV-C in the parasite’s body probably reflects that most of TcTASV-C produced is delivered to the secretory pathway.

### Secretion profile of TcTASV-C in other *T*. *cruzi* strains

**Fig 4:**
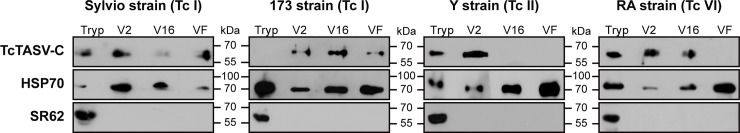
TcTASV-C is secreted in EVs in different *T*. *cruzi* strains. Purified EVs from SylvioX10 (TcI), 173 (TcI), Y (TcII), and RA (TcVI) strains were processed as described in Fig 3. Tryp: trypomastigote; V2: large EVs; V16: small EVs; VF: vesicle-free fraction. The amount of proteins in EVs of different strains was similar, as determined by micro-BCA assay. One representative of three independent experiment is shown.

We then analyzed the secretion profile of TcTASV-C in *T*. *cruzi* strains that encompass a wide spectrum of virulence and also represented the major *T*. *cruzi* lineages [24]. Overall, TcTASV-C was secreted in EVs in all the strains analyzed (Fig 4). Only mild differences in TcTASV-C secretion profile were detected among strains. In the low-virulent SylvioX10 strain, TcTASV-C seemed to be poorly represented in small (V16) EVs, while in 173 strain (DTU TcI)–middle-virulence- the secretion profile of TcTASV-C was quite similar to that found in CL Brener strain (Fig 3), which is also of intermediate virulence in the murine model. In culture-derived trypomastigotes from highly virulent strains (i.e. Y, TcII and RA, TcVI) a more dynamic pattern of secretion was observed, and TcTASV-C was detected alternatively in different fractions (Fig 4 and S3 Fig). Particularly for the Y strain, trypomastigotes released on the 1^st^ day after cells began to lyse usually showed the profile depicted on Fig 4, while TcTASV-C expression shifted to V16 and VF fractions, on EVs secreted from parasites harvested the 2^nd^ and 3^rd^ day, showing a dynamic expression pattern (S3 Fig). As a whole, in all the analyzed strains, TcTASV-C was significantly more represented in the secreted than in the parasite associated fraction. Of note, the total protein content secreted in EVs was similar for all strains thus allowing to discard that changes in TcTASV-C expression by each parasite strain had a correlation with the amount of total EVs secretion.

### TcTASV-C expression and secretion in bloodstream-derived trypomastigotes

Strikingly, the first proteomic evidences of TcTASV-C were registered few years ago with the publication of the proteome of bloodstream trypomastigotes [17]. In contrast, no evidence of expression of TcTASV-C was noticied when proteomics of culture-derived trypomastigotes were analized. Therefore, we decided to investigate the TcTASV-C expression profile in bloodstream trypomastigotes from different *T*. *cruzi* strains, in connection with its expression on culture-derived trypomastigotes.

**Fig 5:**
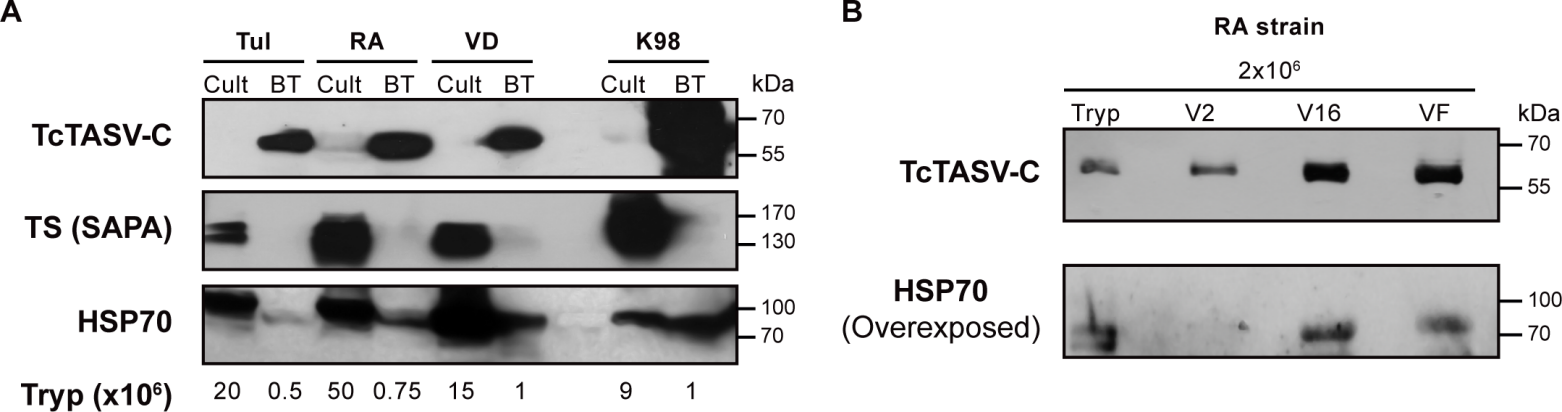
**TcTASV-C expression and secretion profile in bloodstream trypomastigotes A.** Expression of TcTASV-C (upper panel), trans-sialidase (TS-SAPA, middle panel) and HSP70 (lower panel) was analysed in trypomastigotes purified from blood (BT: bloodstream) along with *in vitro* cell-derived trypomastigotes (Cult) from the same strains. Tulahuen (Tul; TcII), RA (TcVI), VD (TcVI), K98 strain (TcI). **B.** TcTASV-C expression on EVs (V2 and V16) and VF secreted fraction from bloodstream trypomastigotes. Tryp: trypomastigote; V2: large EVs; V16: small EVs; VF: vesicle-free fraction.

TcTASV-C was detected in 1x10^6 (or even less) bloodstream parasites (Fig 5A, upper panel, BT lanes) while it was necessary to load more than tenfold of culture-derived trypomastigotes to be weakly detected (Fig 5A, Cult lanes, upper panel). This finding was observed in all *T*. *cruzi* strains assayed, belonging to different lineages, and proved that TcTASV-C is upregulated in bloodstream forms. Importantly, this differential expression pattern between both types of trypomastigotes was not observed with other proteins of *T*. *cruzi*, which were detected accordingly to the parasite amount loaded on the gel (Fig 5A, middle and lower panels). As well as detected for culture-derived trypomastigotes, bloodstream trypomastigotes were also able to strongly secrete TcTASV-C. In the highly virulent RA strain (TcVI) TcTASV-C was essentially identified in all secreted fractions, even from 1-2x10^6 trypomastigotes (Fig 5B and S5 Fig).

### TcTASV-C interacts with the surface of mammalian cells but does not participate in parasite invasion *in vitro*

**Fig 6:**
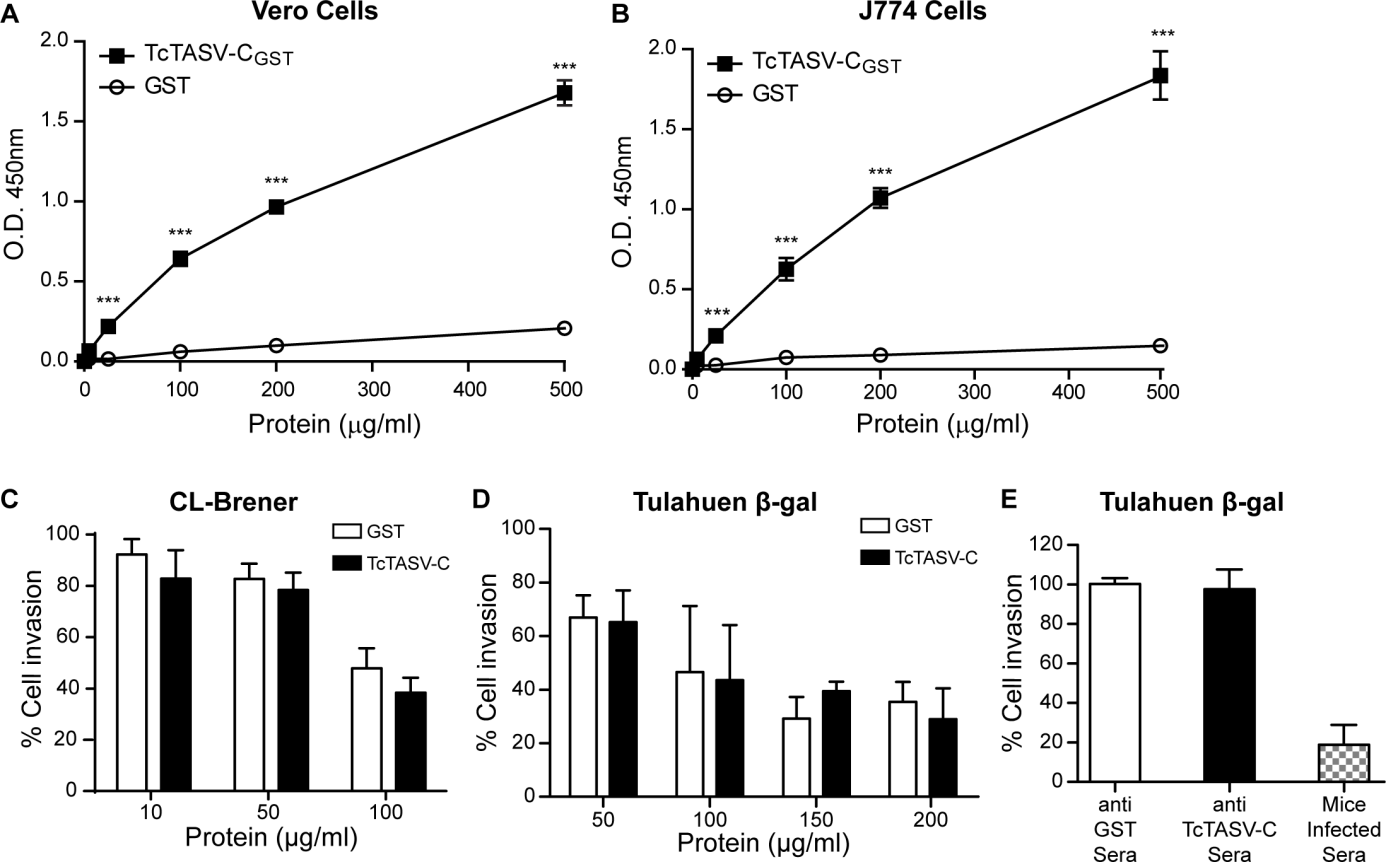
TcTASV-C interacts with mammalian cells but not interfere with *T*. *cruzi* cellular infection. TcTASV-C_GST_ (black squares) or GST (open circles) were incubated with non-phagocytic professional cells (Vero cells) **(A)** or with professional phagocytic cells (J774) **(B).** Binding was assessed with a polyclonal anti-TcTASV-C_GST_ sera, in an ELISA-like assay. Values are means ± standard deviation of one assay (run by triplicate) that is representative of 3 independent experiments. (***p< 0.005 vs GST; Student’s t test). (**C)** Vero cells were pre-incubated with rTcTASV-C for 30 min and then infected with CL-Brener trypomastigotes O.N and washed. After 48 hs cells were fixed and stained with May-Grünwald Giemsa. Infected cells were enumerated by microscopy. **(D)** Cells were treated as in C, and infected with purified bloodstream trypomastigotes (Tul-β-gal) for 18 hs. Then cells were washed and cultured for 72 h. β-galactosidase activity was measured with CPRG, after cell lysis. **(E)** Alternatively, purified bloodstream trypomastigotes (Tul-β-gal) were pre-treated with anti TcTASV-C, anti-GST or sera from infected mice for 30 min, before cell infection. Cell infection and measurements were as in D. Data were normalized to untreated control group, considered as 100% of infection.

As molecules released by the parasite could potentially interact with host cells, we evaluated this possibility for TcTASV-C on Vero (Fig 6A) and J774 (Fig 6B) cells. TcTASV-C (but not the control protein GST) exhibited a dose-dependent adhesive capacity, suggesting a ligand-receptor interaction. Similarly, EVs derived from trypomastigotes also interacted with mammalian cells (S4 Fig). This interaction was only observed with freshly isolated EVs, but not with EVs that had been previously purified and stored at -80°C. We also evaluated a potential role of TcTASV-C on *T*. *cruzi* cell infection, employing as model two *T*. *cruzi* strains from different lineages and obtained from *in vitro* cultures (CL Brener, Fig 6C) or purified from blood of infected mice (Tulahuen strain, expressing β-galactosidase, Fig 6D and 6E) [25,26]. Neither pre-incubation of recombinant TcTASV-C with mammalian cells before infection (Fig 6C and 6D) nor pre-incubation of trypomastigotes with anti-TcTASV-C sera (Fig 6E) interfered with parasite internalization or cellular infection.

### Delivery of antigens in EVs is a *T*. *cruzi* immune evasion strategy

**Fig 7:**
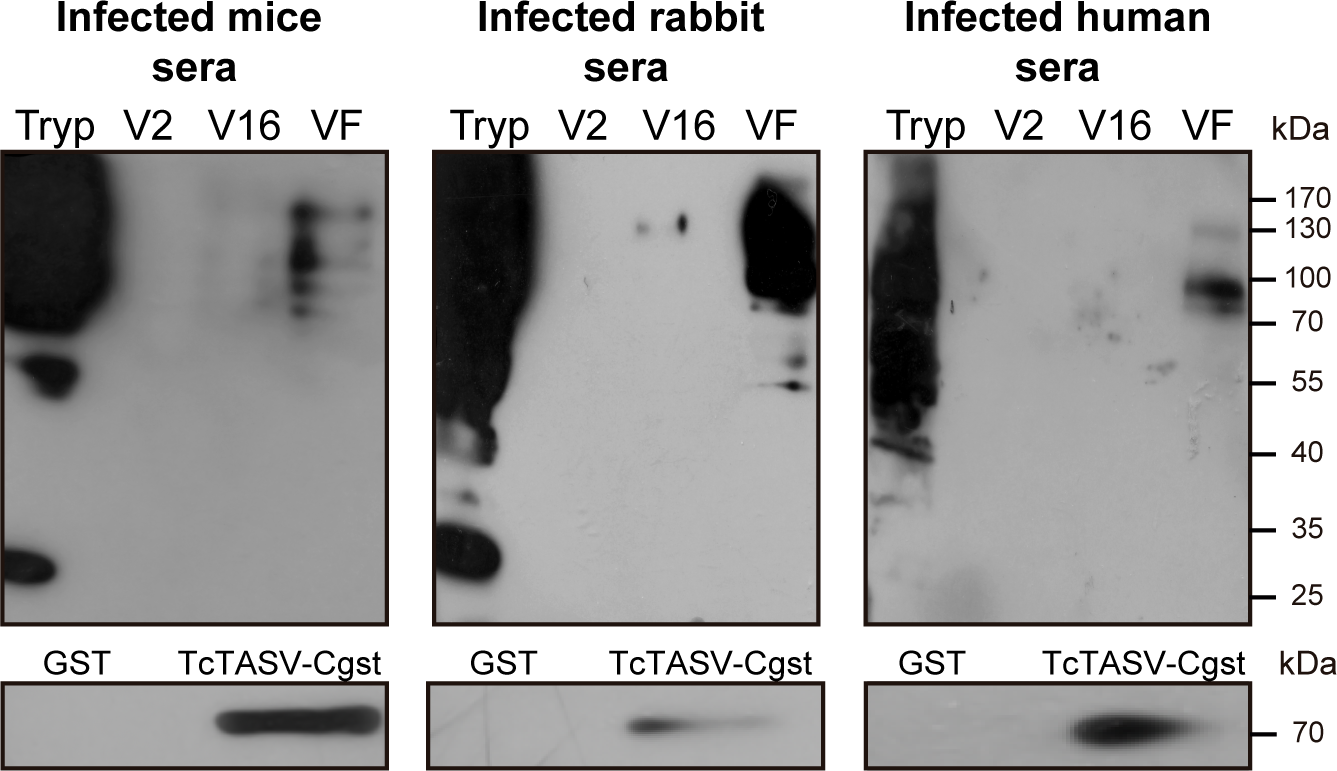
*T*. *cruzi* evades the immune system through the secretion of proteins into EVs. Western blot of CL Brener EVs probed with sera from Infected mice, rabbits and humans. The reactivity of these sera against rTcTASV-C is shown in the bottom panels. Tryp: trypomastigote; V2: large EVs; V16: small EVs; VF: vesicle-free fraction.

The delivery of molecules in vesicles is a well known immune evasion mechanism exploited by parasites [27]. We therefore investigated whether infected hosts could recognize the protein content of secreted vesicles (Fig 7). Pooled sera from humans, mice or rabbits chronically infected with *T*. *cruzi* failed to efficiently detect EVs antigens, although they strongly reacted with trypomastigote antigens and with “naked” secreted proteins, both from RA and CL Brener strains (Fig 7 and S6 Fig). These findings led us to hypothesize that immunization with TcTASV-C–which is highly expressed in bloodstream trypomastigotes and also secreted- would be a good target for immunotherapy control. This hypothesis was encouraged by our previous finding of a TcTASV-C gene fragment among a pool of protective antigens [12]. Besides, being TcTASV-C expressed at early stages of the infection [10], we hypothesize that humoral immune response could be mediating TcTASV-C neutralization.

### TcTASV-C immunized animals presented a strong humoral response and a partially delayed appearance of bloodstream trypomastigotes

To evaluate the performance of TcTASV-C as vaccine antigen, we designed a DNA-prime protein-boost schedule of immunization. The first 2 doses consisted of plasmid DNA of an eukaryotic expression vector carrying a fragment of TcTASV-C [12] adjuvanted with a plasmid coding for GM-CSF [28]. In the 3^rd^ and 4^th^ doses, mice were boosted with TcTASV-C recombinant proteins (two different genes fused to GST or histidine tags, rTcTASV-C_GST_ and rTcTASV-C_HIS_) adjuvanted with aluminium salts.

**Fig 8:**
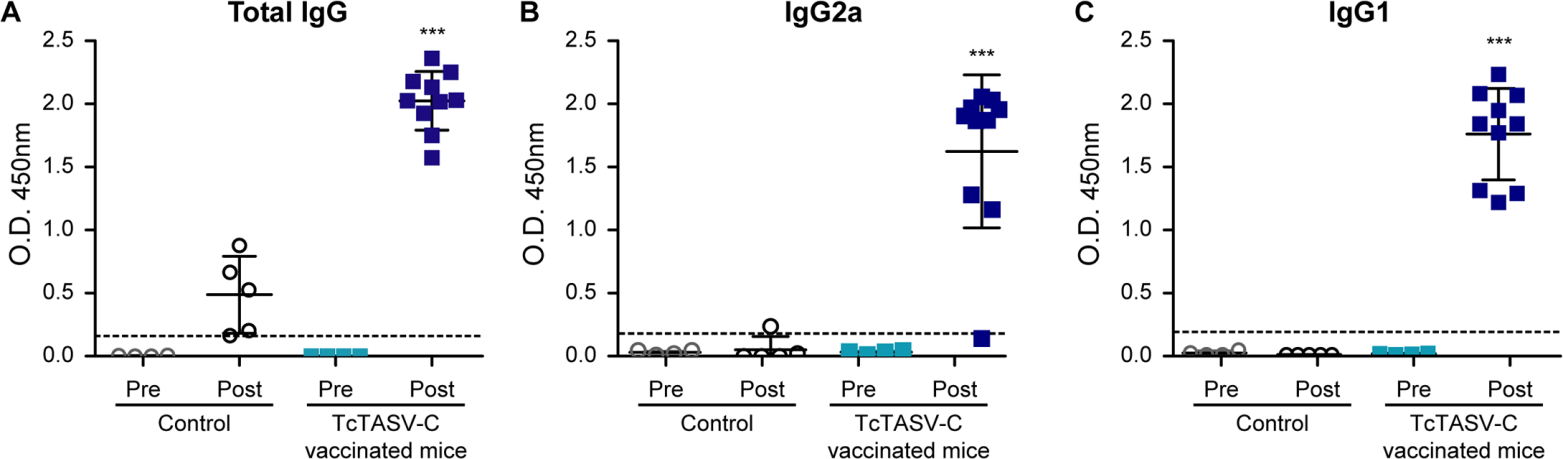
Anti-TcTASV-C antibody response in mice after vaccination. Serum samples were obtained before the first (Pre) and 15 days after the last dose (Post) of immunization with TcTASV-C or Control schemes. **(A)** Total IgG, **(B)** IgG2a and (**C**) IgG1 responses against rTcTASV-C_GST_ were determined by ELISA. Each dot represents one individual animal. Absorbance against GST was subtracted. Dotted line indicates the cut-off (***p< 0.005 vs control group, one-way ANOVA).

As expected, immunization was effective to induce high levels of total anti-TcTASV-C IgGs (Fig 8). Most animals presented a mixed Th1/Th2 response, with strong IgG2a and IgG1 responses (Fig 8B and 8C). However, the cellular and cytokine response in splenocytes obtained from mice 15 days after immunization, showed a low proliferative response and negligible levels of IFN-ɣ and IL-10 after rTcTASV-C restimulation in culture.

**Fig 9:**
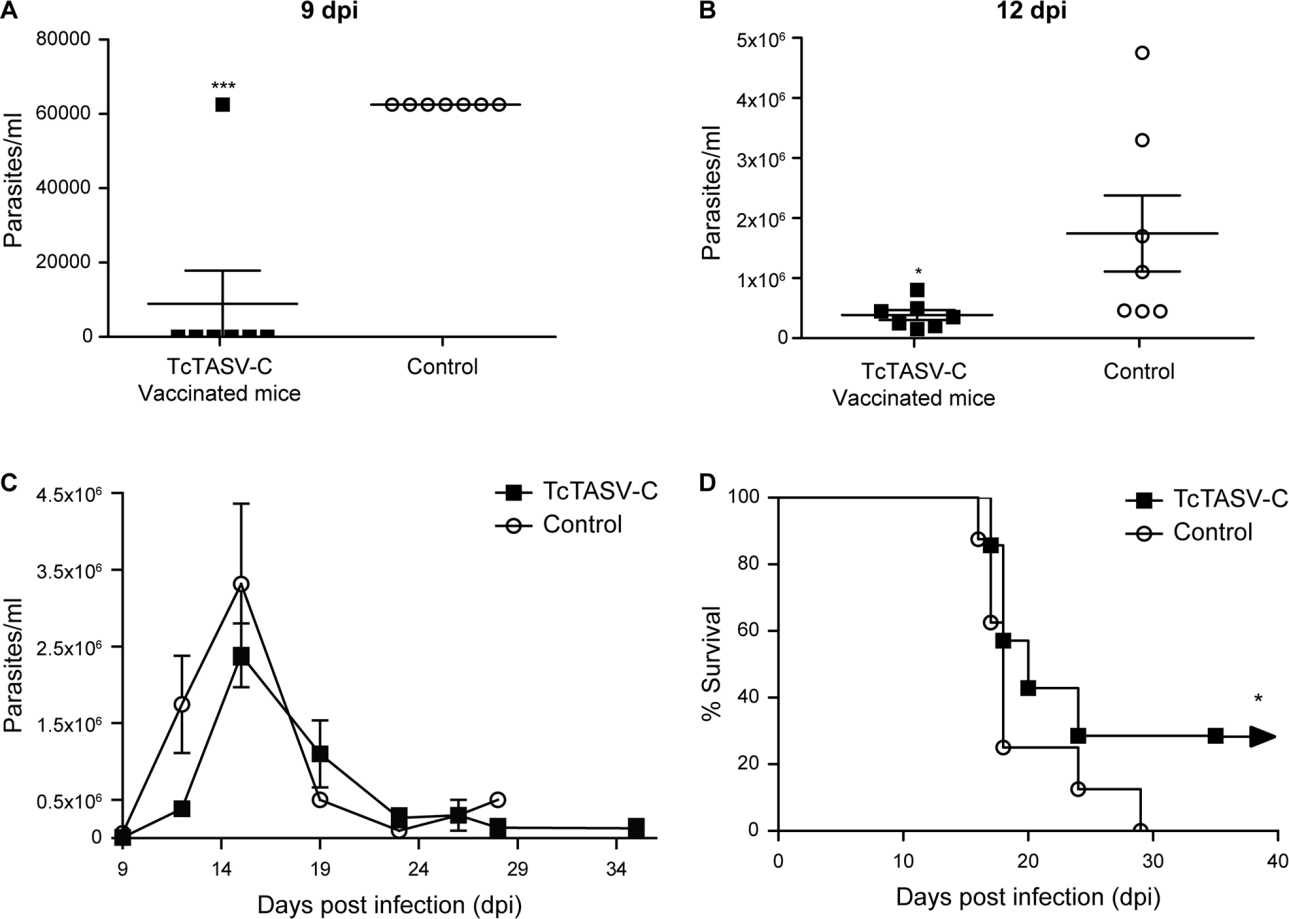
Parasitological outcome of TcTASV-C vaccinated mice challenged with a virulent *T*. *cruzi* strain. Groups of mice (n = 5) were immunized with TcTASV-C or vehicle (Control) and challenged with *T*. *cruzi* trypomastigotes of the RA strain (TcVI) 15 days after the last dose. Animals were followed up for parasitemia and survival. Parasitemia of individual mice at 9 (**A**) and 12 (**B**) days post-infection. (**C)** Parasitemia during the course of infection in TcTASV-C vaccinated and Control groups (*p<0.05 and ***p<0.005 vs control; Mann-Whitney test). (**D)** Survival curve of challenged TcTASV-C vaccinated and Control groups (**p<0.05 vs Control group; Log-Rank test).

Two weeks after the last dose, animals were challenged with parasites of the highly virulent RA strain (DTU TcVI). TcTASV-C vaccinated mice exhibited a delayed appearance of circulating trypomastigotes and lower parasitemia peaks (Fig 9A–9C). Bloodstream trypomastigotes were detected from the day 9^th^ on, in all controls while were nearly unnoticeable until day 12^th^ in all TcTASV-C vaccinated (Fig 9A–9C). Besides, TcTASV-C vaccinated mice presented reduced trypomastigote numbers at the peak of parasitemia (Fig 9C) and–overall- lower bloodstream parasite levels than the control group (Fig 9C; p<0.05, at 9 and 12 pdi, Mann-Whitney test). Immunized mice also showed higher survival rates than controls (Fig 9D; TcTASV-C, ~30%; control group, 0%; p<0.05 Log-Rank test). All animals that survived at the end of experiments belonged to TcTASV-C vaccinated groups.

**Fig 10:**
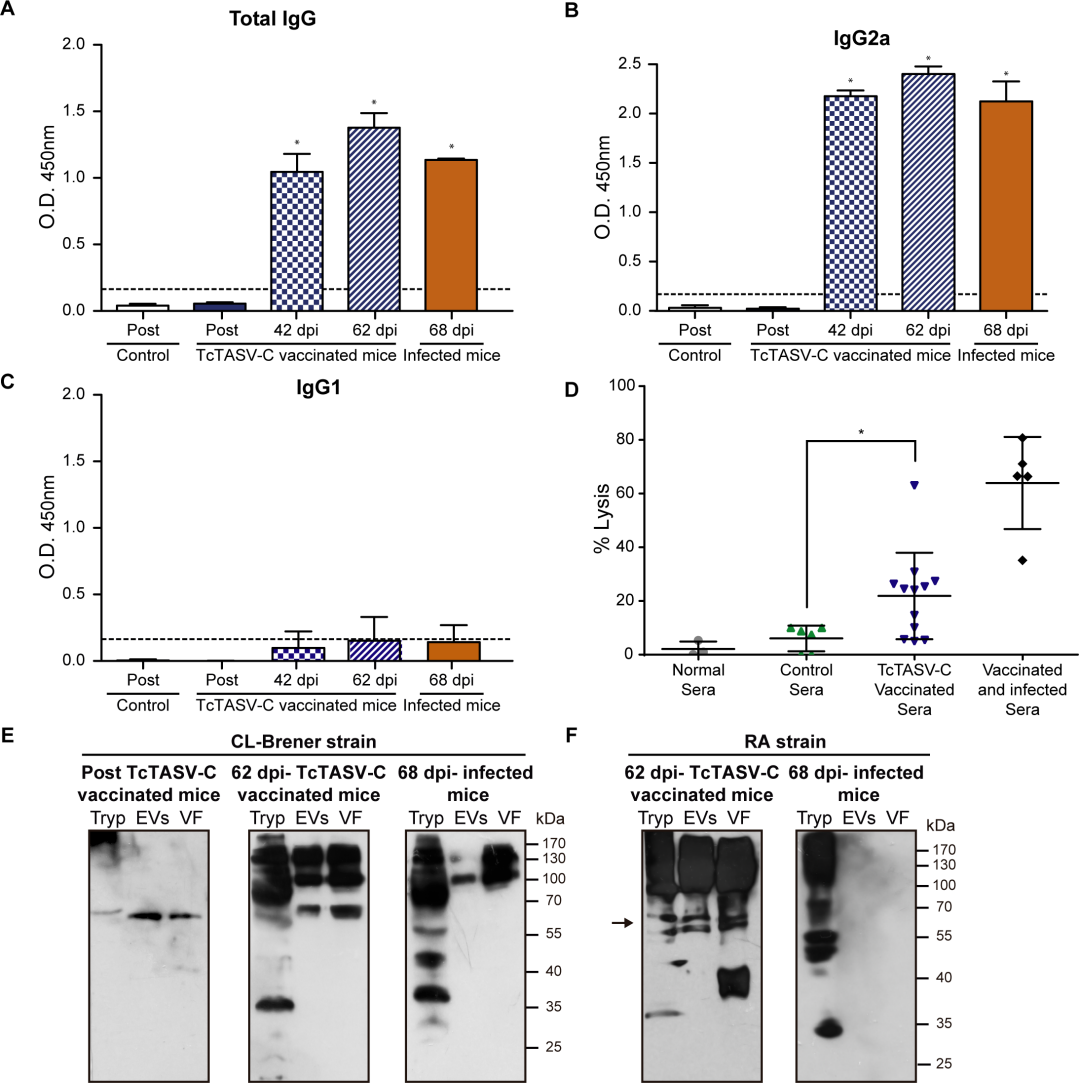
Humoral anti-*T*. *cruzi* response after vaccination and after *T*. *cruzi* challenge. Serum samples were obtained before the first (Pre), 15 days after the last dose (Post) of immunization and at 42 and 62 dpi. A pool of sera from chronically infected (non-vaccinated) mice was also evaluated (68 dpi infected mice, spontaneous survivors from infection with 100 RA trypomastigotes by the intradermal plantar route). **(A)** Total IgG, **(B)** IgG2a and **(C)** IgG1 antibodies against total *T*. *cruzi* antigens were determined by ELISA. Dotted line indicates the cut-off value. (**D)** Complement-mediated lysis of *T*. *cruzi* trypomastigotes. Parasites (500000/assay) were incubated with inactivated sera from preimmune (Normal sera), GST-vaccinated (Control group), TcTASV-C vaccinated or vaccinated and surviving mice for 1 h at 37°C, followed by treatment with fresh human complement (1:4) or inactivated human complement for an additional 1 h at 37°C. Trypomastigote lysis was calculated by counting living, motile and unstained parasites in a Neubauer chamber after staining with Trypan blue. Each dot represents 2–3 pooled sera, assayed together. **(E)** and **(F)** Western blot of trypomastigote lysate (Tryp), EVs and EV-free supernatant fraction (VF) from CL-Brener **(E)** or RA **(F).** Proteins were probed with immune sera from vaccinated mice (Post TcTASV-C vaccinated mice), sera from surviving vaccinated mice (62 dpi-TcTASV-C vaccinated mice) and RA-infected (unvaccinated) mice (68 dpi-Infected mice). *p<0.05 when pairwise compared to Control group by One-way ANOVA.

After *T*. *cruzi* challenge, vaccinated mice showed a strong response against parasite antigens, with an IgG2a-biased isotype, similar to that found in infected animals (Fig 10A, 10B and 10C). Besides, sera from vaccinated mice were able to lyse trypomastigotes in the presence of an external complement source (Fig 10D); the lytic response was increased in mice after infection. Focusing on the anti-EV response after *T*. *cruzi* challenge, vaccinated animals reacted not only with TcTASV-C but also with other EV antigens, that were unseen by infected but non-vaccinated animals (Fig 10E and 10F). Therefore, the vaccination was able to trigger an EV-focused immune response after challenge that can’t be mimicked by unvaccinated infected animals.

### Mapping of B-cell epitopes in TcTASV-C with sera from protected mice

**Fig 11:**
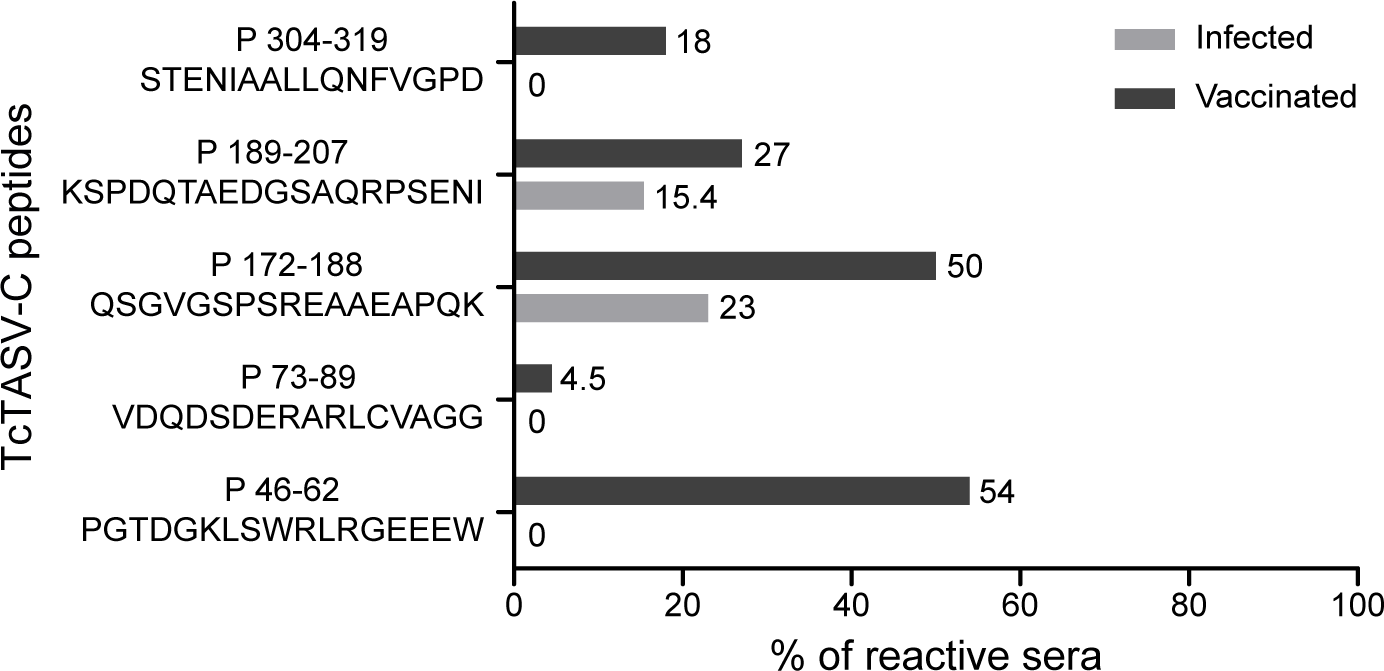
B-cell epitopes detected in TcTASV-C by sera from vaccinated mice. Sera from vaccinated (uninfected, n = 22) or infected (unvaccinated and TcTASV-C reactive, n = 13) mice recognized differential TcTASV-C peptides, evaluated in an ELISA format. Reactivity is expressed as % of sera that were reactive for each peptide. All sera were reactive against TcTASV-C proteins. Numbers in peptides indicate the amino acids of TcTASV-C encompassed by the peptide (i.e. P46-62, P172-188).

As the main immune response detected in immunized animals was humoral, we mapped the TcTASV-C epitopes detected. Peptides covering putative TcTASV-C epitopes were designed by weighing linear B-cell epitope predictions (bioinformatic approach) and those epitopes previously discovered in a high-density peptide microarray screened with human sera [29]. We selected 5 peptides of 15–20 amino acids to evaluate the reactivity of sera from TcTASV-C vaccinated, control and infected unvaccinated mice (Fig 11). Eighty-six percent (86%; 19/22) of the sera from vaccinated mice reacted with at least one peptide, and 45% reacted with 2 or more peptides (S1 Table). In contrast, only 30% (4/13) of sera from unvaccinated infected mice (with previously reported reactivity against TcTASV-C) recognized any of these peptides (S1 Table).

P46-62 and P172-189 were the peptides most detected by the sera from vaccinated group, with 84% (16/19) of sera reacting with one or both of them (Fig 11 and S1 Table). P46-62 was the peptide most detected by sera from vaccinated mice while it was not detected by any of the sera from the infected group. Interestingly, P46-62 is part of the *tasv_all* motif, but is only partially present in the rTcTASV-Cs employed in the vaccination schedule (TcTASV-C_HIS_: KLSWRLRGEEEW; TcTASV-C_GST_: SWRLQGEEEW). Even more striking, the reactivity to peptide P46-62 seems to be driven by the RLR triplet or the second arginine, since an identical peptide with an unique substitution that changes the RLR motif to the RLQ, turned it into an unrecognized peptide (P47-63; S1 Table). Both RLR and RLQ sequences are present in TcTASV-C genes (see Fig 1), and represented by the rTcTASV-Cs employed in the vaccination scheme (RLR in TcTASV-C_HIS_, RLQ in TcTASV-C_GST_).

Altogether these results support the idea that the broad anti-peptide reactivity of immunized mice is probably mediating the partial resistance and/or the delay in the appearance of circulating trypomastigotes in challenged mice.

## Discussion

The *T*. *cruzi* TcTASV gene family remained unobserved until a few years ago when it was identified by our group through a trypomastigote-enriched cDNA library [9]. Almost simultaneously, an expression library immunization approach designed to discover novel vaccine antigens in *T*. *cruzi*, spotlighted the TcTASV-C subfamily, because a fragment of a TcTASV-C gene was identified in a pool of protective clones [12]. A distinctive feature that characterizes TcTASV proteins–and particularly the TcTASV-C subfamily- is their predominant expression in bloodstream trypomastigotes. Recent transcriptomic and proteomic studies uphold our previous observations that the TcTASV family is over-represented in the trypomastigote stage [17,18], and therefore could represent an interesting target for rational intervention in *T*. *cruzi* infection.

Here the TcTASV-C expression and secretion dynamics and its performance as an individual vaccine candidate were analyzed. We demonstrate that, despite its scarce expression on culture-derived trypomastigotes, TcTASV-C is strongly secreted, and is a major component of trypomastigote’s EVs, at least in the *T*. *cruzi* reference strain CL Brener. This was observed both by western blot and proteomics on large (V2) and small (V16) EVs. It is a novelty, although not unexpectedly, that a parasite-associated and low-expressed protein (or protein family) is actually a highly abundant component of the trypomastigote secretome. The secretion of EVs by parasites has been proposed as a pathogen-driven mechanism aimed to generate -in the host- an environment that favours the initial infection [30–34]. Indeed, in most of the tested *T*. *cruzi* strains, TcTASV-C was mainly secreted contained into EVs. Of note, the more virulent strains (i.e. RA and Y) presented also a more dynamic secretion pattern (Fig 4, Fig 5, S3 Fig and S4 Fig). On the other hand, we have shown that TcTASV-C expression is upregulated in bloodstream parasites, suggesting that some molecules present in the host trigger TcTASV-C expression.

The potential of TcTASV-C as an individual vaccine candidate, however, was somehow limited to the acute phase. In our model, TcTASV-C immunized mice achieved an enhanced control of parasitemia at the beginning of the infection. The delayed appearance of bloodstream trypomastigotes was along with the presence of functional antibodies in sera from TcTASV-C vaccinated mice, with ability to lyse trypomastigotes by ADCC. This is also consistent with the detection of TcTASV-C early upon infection and suggests that TcTASV-C could have a role during this phase of infection [10,13] (unpublished results). We hypothesize that the window of time with lower bloodstream parasites, gives a handicap to TcTASV-C primed mice to launch effector mechanisms against the parasite. However, it has to be said, the humoral response induced by vaccination was not strong enough to completely protect and clear parasites from a lethal challenge, and mortality rates were only mildly improved. Likewise, Ramirez *et al* (2017) [35] have recently reported that EVs derived from the interaction between mammalian cells and trypomastigotes potentiated parasitemia, particularly in the early acute phase (3–6 days) of infection. This effect was stage-specific since it was not observed with EVs derived from the interaction of mammalian cells with metacyclic trypomastigotes or epimastigotes, suggesting that stage-specific EVs components might play a role in survival and dissemination of this parasite stage in the vertebrate host [35].

Secretion of virulence factors contained in extracellular vesicles has also been understood as a parasite strategy to deliver long distance effector molecules that should act in concert [36]. In particular, *T*. *cruzi* trypomastigotes release EVs that can interact with the host and modulate immune responses. The first communication in this way was in 2009, when Trocoli-Torrecilhas *et al* [37] demonstrated that inoculation of mice with naked extracellular vesicles predisposed them to a more virulent infection, along with a strong inflammatory tissue damage and higher parasitic loads in heart. In fact, the effect observed with whole EVs had been observed several years before with an EV cargo molecule, the trans-sialidase (TS). Chuenkova and Pereira (1995) [38] reported that mice sensitized with TS were more susceptible to *T*. *cruzi* infection, displaying enhanced parasitemia and mortality. Here, by analyzing the proteome from CL Brener EVs, we found peptides of both TS and TcTASV families, suggesting that both components of trypomastigotes are secreted as part of the same cargo and can act in a concerted fashion. Actually, in retrospective, we found several EV cargo proteins employed as vaccine antigens with promising results [12,39–46]. Interestingly, peptides of most of these proteins were found in our EV proteome (Tc24, SA85, CRP, MASP, TS, tryparedoxin-peroxidase, paraflagellar rod proteins, etc). We propose that immunization with some of the molecules delivered into EVs with proper adjuvanticity, could allow the host to develop an adequate immune response against *T*. *cruzi*.

The prime and boost vaccination scheme employed here mostly triggered a humoral mediated immune response able to block or neutralize surface anchored and/or secreted TcTASV-C. Although yet unknown, we speculate that the possible function of the TcTASV-C subfamily is exerted through its most conserved motif (*tasv_c*), which encompasses a 50 amino acid long sequence at the amino terminus of the protein. Also, the shorter *tasv_all* motif common to all TcTASV subfamilies can be found within the *tasv_c* motif, but with specific amino acids at certain positions for each subfamily (see Fig 1). Interestingly, a linear B-cell epitope located within the *tasv_all-tasv_c* motif was exclusively recognized by sera from TcTASV-C vaccinated mice (P46-62, Fig 11). This reactivity seems to be specifically prompted by the prime and boost vaccination scheme since sera from infected unvaccinated mice are unable to react with this peptide, suggesting that antibodies against this motif are mediating the TcTASV-C neutralization achieved–at least partially- by this vaccination scheme during the early infection.

Packaging molecules into EVs can also be considered as a parasite driven strategy to escape from the host immune surveillance or extracellular degradation until they reach the target cells or tissues. In our hands, EV proteins contained in trypomastigote-secreted EVs were not detected by sera from infected hosts from different species, in contrast with trypomastigote-associated and freely secreted antigens, that were recognized by sera from infected hosts. Indeed, this finding suggests that ~30% of sera from infected hosts that do recognize TcTASV-C actually reacted against proteins attached to the parasite’s surface or freely-secreted to the environment, but not against the TcTASV-C genes that are secreted contained into EVs. We support the hypothesis that secretion of cargo in EVs (and particularly the secretion of TcTASV-C) is another parasite-driven immune evasion mechanism. In a recently published work, Bautista-Lopez *et al* (2017) [14] looked for “Trypomastigote Excreted Secreted Antigens” (TESA, because the whole secreted population was analyzed) that are exposed to the host immune system. They carried out an immune capture assay with *T*. *cruzi*-infected patient’s antibodies to screen for novel and secreted antigens, which could be useful markers of disease status. In accordance with our results, and although TcTASV-C peptides were found in the TESA proteome, none of TcTASV proteins were revealed by patient’s sera. Altogether, these results reinforce the idea that most of the proteins delivered into EVs are hidden from the host or, at least, are hard to be detected in the way they are presented to the host immune system.

In 2016, Queiroz *et al* [15] published the first proteomic analysis of the trypomastigote secretome (which included both free and EV-secreted proteins) from the Y strain (DTU TcII), and soon after Bautista Lopez *et al* (2017) [14] presented the proteome of EVs derived from the culture of both cells and trypomastigotes (Tulahuen strain; DTU TcVI). In both of these proteomes TcTASV peptides were eventually identified, supporting the results presented here that demonstrated that, despite being a medium-size gene family, TcTASV proteins are an important component of the trypomastigote secretome and EVs. Regarding the expression of TcTASV-C in the trypomastigote EVs, it is notable that TcTASV-C is easily detected by western blot, suggesting it as a major EV component, especially in contrast with its weak expression on parasite’s body of culture-derived trypomastigotes. Although TcTASV-C is hard to be detected in culture-derived trypomastigote homogenates (undetectable for the conditions of western blot in Fig 3B, upper panel), it is revealed as a major component of EVs in CL Brener strain. In fact, the identification of peptides from 4 different TcTASV-C genes in our EV proteome corresponds with this observation and also with the 2D gel results, where 4 spots were detected as TcTASV-C proteins in the secreted fraction. The picture obtained for TcTASV-C could indicate that the paucity of TcTASV-C in the parasite’s body probably reflects that most of the protein produced is delivered to the secretory route, thus suggesting that its putative function is related somehow to the development of a permissive environment for early *T*. *cruzi* settlement. It is well known, but poorly documented, that parasites–and particularly *T*. *cruzi* trypomastigotes- express a very different set of molecules when isolated from the host (i.e. *in vivo* infection) than from culture (i.e. *in vitro* infection), basically in response to the pressure of the immune system. Here, we demonstrate that TcTASV-C expression is much higher in bloodstream than in culture-derived trypomastigotes. We show that this is true for different *T*. *cruzi* strains and also that it is specific for TcTASV-C, but not for other antigens or virulence factors of trypomastigotes. We still do not known what factors of the vertebrate host trigger this expression, which is a matter of our current research. Besides, these results highlight the relevance of working with trypomastigotes obtained from *in vivo* sources to study the *T*. *cruzi* biology, especially when research involves parasite stages that are under immune system pressure in the vertebrate host.

The delivery of virulence factors in exosomes or extracellular vesicles is also a strategy to interfere with host cell signaling pathways required to control infection. Exposure of mice to exosomes of *L*. *infantum* resulted in higher parasitic loads in spleen, which was linked to a suppressive T cell phenotype [30,31]. As well as *Leishmania* exosomes display immunomodulatory properties, *T*. *cruzi* extracellular vesicles also do. In fact, Nogueira *et al* (2015) [47] found that different *T*. *cruzi* strains secreted different concentration of vesicles. This variability could not be associated with the current *T*. *cruzi* DTU classification because -for example- two TcI strains presented polar secretion levels (Col vs. YuYu). Protein concentration and alpha-galactosyl residues in secreted EVs also varied among the different strains, and without a lineage specific association [47]. Focusing on the modulation of immune responses by EVs in the acute phase of infection, only after stimulation with EVs from YuYu (DTU TcI) and CL-14 (DTU TcVI), peritoneal macrophages from C57BL/6 mice produced high levels of proinflammatory cytokines (TNF-alpha) and NO, via the TLR-2. This profile was not stimulated by EVs from other *T*. *cruzi* strains. Similar findings were recently reported by Clemente *et al* (2017) [48] employing EVs secreted by metacyclic trypomastigotes from other strains. In the present work, we registered a variable expression of TcTASV-C in the different secretory fractions (i.e. V2, V16 and soluble factors) among the different strains analyzed. Although we found similar levels of total protein content in EVs derived from the different strains, it should be stated that the protocols employed to isolate EVs and the strains analyzed were different. As in previous works, we could not link a particular secretion profile with a certain *T*. *cruzi* DTU or strain. This complex scenario led us to speculate that differences in EVs cargo could reflect the broad spectrum of clinical manifestations observed in Chagas’ disease. Our opinion is that we are still building a puzzle from somehow complementary but still fragmented data, showing currently a complex and not very well understood picture.

In brief, we have demonstrated that TcTASV-C is a major component of bloodstream trypomastigotes, and that TcTASV-C is mainly secreted, either contained into EVs or free. Besides, although with the prime and boost strategy employed TcTASV-C did not result a promising vaccine candidate, it was possible to interfere with the early acute phase of *T*. *cruzi* infection. Indeed, the strong anti-TcTASV-C humoral immune response elicited by immunizations allowed to understand–partially- the TcTASV-C functionality; we hypothesize that TcTASV-C is involved in the establishment of the initial *T*. *cruzi* infection in the mammalian host. Although we highlight TcTASV-C as a potential antigen to bit the parasite in the early acute phase, we bear in mind that an effective vaccine to control Chagas’ disease should include other antigens and/or trigger also other arms of the host immune response. Ultimately, results presented here strongly highlight TcTASV-C as a novel secreted virulence factor of *T*. *cruzi* trypomastigotes.

## Materials and methods

### Ethical statement

All protocols conducted with animals were designed and carried out in accordance with international ethical standards for animal experimentation *(Helsinki Declaration and its amendments*, *Amsterdam Protocol of welfare and animal protection and National Institutes of Health*, *USA NIH*, *guidelines)* and were approved by the Institutional Animal-Care Ethics Committee of the University of Buenos Aires (CICUAL, res number: 2846/2013) and from University of San Martin (CICUAE, protocol number: 01/2012 and 08/2016).

### TcTASV-C recombinant proteins

TcTASV-C_GST_ (amino acids 65 to 330 of ORF *Tcruzi_1863-4-1211-93*) was already cloned in our laboratory in pGEX-3X and was expressed and purified as we previously described [10]. The same procedure was used with GST.

Amino acids 52 to 342 of the TcTASV-C gene AM492203 (GenBank; emb.CAM33606.1) were cloned between BamHI and KpnI restriction sites, fused in the N-term to a Histidine tag into pQE-30 (Qiagen). A point mutation was introduced to change the amino acid H56R. TcTASV-C_HIS_ was expressed and purified by standard methodologies for histidine-tagged proteins (The QIAexpressionist). Purity of proteins was analyzed by SDS–PAGE, followed by staining with Coomassie Brilliant Blue. Proteins were quantified (Bradford assay and/or Picodrop) and dialyzed against PBS. Recombinant proteins were stored aliquoted at −80°C until use.

For mice immunizations, purified recombinant proteins were incubated with a Polymyxin B resin in a column format (Detoxy-Gel Endotoxin Removing Gel Thermo Scientific). Endotoxin levels were quantified by Amebocyte lysis assay (Limulus Amebocyte Lysate Test, Lonza). Only preparations with endotoxin levels <100 U/mg were used.

### Anti-TcTASV-C antisera

Recombinant TcTASV-C_GST_ was digested with Factor Xa (GE Healthcare) and the purified TcTASV-C fragment was used to produce specific anti-TcTASV-C sera in mice [49]. The specificity of the anti-TcTASV-C sera was verified by competition assays and western blot, both against trypomastigotes lysates and recombinant proteins.

Recombinant TcTASV-C_GST_ was used to produce complete anti-TcTASV-C-GST serum in mice, following the same immunization protocol described above. The sera obtained reacted both with TcTASV-C and GST.

### Parasites and cell cultures

Vero and J774 cells were grown at 37°C in a 5% CO_2_ humidified atmosphere in MEM or RPMI (Gibco), respectively, supplemented with 10% fetal bovine serum (Natocor), 10 μg/mL streptomycin (Sigma), 100 U/mL penicillin (Sigma).

Cell-derived *T*. *cruzi* trypomastigotes were cultured by passages in Vero cells at 37°C and 5% CO2 humidified atmosphere in MEM (Gibco Life Technologies) supplemented with 10% fetal bovine serum, 10 μg/mL streptomycin, 100 U/mL penicillin. Trypomastigotes were harvested from supernatants of infected cells as previously described [10]. As a rule, *T*. *cruzi* stocks are kept in liquid nitrogen and all strains are regularly thawed twice a year to preserve their biological characteristics. Parasites from Sylvio (TcI), 193–173 (TcI), K98 (TcI), Y (TcII), Tul (TcVI), VD (TcVI), CL Brener (TcVI) and RA (TcVI) were employed [50–54].

Bloodstream trypomastigotes of the RA strain (DTU TcVI) were maintained *in vivo* by weekly passages in CF1 mice with 10^5^ trypomastigotes, at IMPaM (School of Medicine, University of Buenos Aires-CONICET) and at the BLS3 laboratory at UNSAM. The Tulahuen strain expressing *E*. *coli* β-galactosidase (Tul-β-gal) was also maintained *in viv*o by passages on CF1 mice at UNSAM [25]. Purification of bloodstream trypomastigotes was essentially carried out by a Ficoll gradient with a swinging bucket rotor, essentially as previously described [55]. Bloodstream RA trypomastigotes used for EV purification, were either purified as stated above or by swimming (2 x 40 min at 37°C), essentially as described by Miranda et al (2015) [56]. Briefly, heparinized blood was diluted with 3 volumes of PBS and, after centrifuged at 300 *x* g for 5 min, the sample was incubated for 40 min at 37°C. The supernatant containing parasite forms was then carefully harvested–to exclude the erythrocyte containing phase–and the procedure was repeated twice. Then, trypomastigotes were pelleted, washed with PBS-1% BSA, resuspended in MEM and incubated for shedding assays, as described below. Similar volumes of blood from non-infected mice were processed in parallel and used as controls.

### Shedding assays and extracellular vesicles (EVs) isolation

Cell-derived trypomastigotes, were washed with MEM without serum and incubated at a concentration of 10^8^ parasites/ml in MEM at 37°C, during 6 hours, in a 5% CO_2_ humidified atmosphere. Trypomastigote-secreted products (soluble plus vesicles) were isolated as previously described [10]. A similar procedure was carried out for bloodstream trypomastigotes.

Extracellular vesicles were purified by an iodixanol density gradient (Optiprep, Sigma) ultracentrifugation as described by van Deun J *et al* (2014) [57] or by sequential ultracentrifugation as described by Bayer-Santos *et al* (2013) [22]. Briefly, for both procedures, after shedding, parasites were removed by centrifugation and the cell-free supernatant filtered through a 0.45-μm syringe filter (Micron Separation Inc.).

A discontinuous gradient was created by layering 2,7 mL of 40%, 20%, 10% and 2,3 mL of 5% Optiprep solutions from bottom to top in a 13,2 mL polyallomer tube (Beckman Coulter). The cell-free supernatant of trypomastigotes (EVs plus free secreted fraction, 2 ml) was overlaid onto the top of the gradient, which was then centrifuged for 18 hours at 100,000 x *g* without brake at 4°C in a SW 41 Ti rotor in an Optima XL 100k ultracentrifuge (Beckman Coulter). Gradient fractions of 1 mL were collected from the top of the gradient. Density was determined weighing on an electronic balance a known volume of each fraction.

Alternatively, to isolate large and small extracellular vesicles, EVs plus the free secreted fraction were centrifuged at 100.000 x *g* for 2 h at 4°C to obtain the first pellet, enriched in large extracellular vesicles (V2), and the resulting supernatant was centrifuged again at 100.000 x *g* for 16 h at 4°C, to obtain the second pellet, enriched in small extracellular vesicles (V16), and the EV-free supernatant fraction (VF). All ultracentrifugation steps were carried out in a 70Ti fixed angle rotor in an Optima XL 100k ultracentrifuge (Beckman Coulter).

All relevant methodological data of our EV’s isolation procedures have been submitted to the EV-TRACK knowledgebase (EV-TRACK ID: EV170020) [58].

### Electron microscopy

Extracellular vesicles were resuspended in Hepes Buffer, pH 6.5, and fixed with paraformaldehyde (4% in Hepes). Negative staining was carried out on grids coated with acrylic membranes and graphene oxide. Extracellular vesicles were stained with 5 μl of 0.5% ammonium molybdate at pH 7.5, and observed using a Zeiss EM 109T transmission electron microscope operating at 80kV; the images were acquired with a Gatan ES1000W (11 Mpx) digital camera.

### 2D SDS-PAGE

For 2D gel electrophoresis, trypomastigotes were incubated in serum-free DMEM for 2 h a 37°C (or at 0°C for controls). The medium containing the secreted antigens and the parasites were separated by centrifugation at 4000 x *g* for 10 min at 4°C. Pelleted parasites were washed twice in 10 mM Tris-Cl, pH 7.0, 25 mM sorbitol and, after being pelleted by centrifugation, were lysed by vortexing for 30 s in 250 μl of IEF rehydration buffer (9M urea, 2M thiourea, 2% CHAPS, 65 mM DTT, 0.5% IPG buffer [Amersham Pharmacia] and 0.002% bromophenol blue) with protease inhibitor cocktail (Roche). The secreted material was also mixed with IEF rehydration buffer and both the trypanosome lysate and the secreted antigens were incubated at R.T for 1 h, with vortexing for 30 s every 15 min, as described by van Deursen *et al* (2003) [59]. Samples were loaded into Immobiline DryStrip (pH 4–7, 13 cm; GE Healthcare) and isoelectric focusing carried out in an IPGphor Isoelectric focusing System for 24 h. Second-dimension SDS-PAGE was carried out in a Hoeffer SE 600, and gels were transferred to nitrocellulose membranes in a semi-dry TE 70 PWR (Amersham Biosciences). Blocking and washing solutions and antibodies used were similar to those described below for conventional western blot.

### Extracellular vesicles (EVs) western blot

Thirty million (30x10^6^) of *in vitro* cell-derived trypomastigotes, or its secretion equivalent from small (V16), large (V2), total EVs or the soluble EV-free fraction (VF) were electrophoresed on 10% denaturing polyacrylamide gels, and transferred to nitrocellulose membranes by standard methodologies [60]. The correct transfer was verified by reversible membrane staining with Ponceau Red (5% w/v) in 1% (v/v) acetic acid. The membrane was blocked with PBS-3% non-fat milk for 1 hour, washed with PBS-0.05% Tween and incubated with primary antibodies. Anti TcTASV-C (mouse, 1/400), anti HSP-70 (rabbit, 1/1000) and anti-SR62 (rabbit, 1/1000) were employed. Then, washes were repeated and membranes were incubated with a peroxidase-conjugated secondary antibody (anti-mouse or anti-rabbit, both from Thermo Scientific) for 1 hour and the washes repeated. For the detection, we used a chemiluminescent reagent (SuperSignal West Pico, or SuperSignal West Femto, Thermo Scientific). The emitted signal was detected by exposure on radiographic plates (AGFA). For bloodstream trypomastigotes, or its secretion equivalent from EVs, an additional blocking step with non-labelled anti-mouse IgG (Sigma-Aldrich) before incubation with the primary antibodies was included. Western blots were developed as indicated above or with Alexa Fluor 590 goat anti-mouse IgG or Alexa Fluor 680 goat anti-rabbit IgG as secondary antibodies (Invitrogen) at a 1:20000 dilution and visualized with an Oddysey Infrared Imager (Li-Cor).

### Proteomic analysis

Purified EVs (20 μg) were diluted in 50 mM ammonium carbonate. Mass spectrometry analysis was carried out at Centro de Estudios Químicos y Biológicos por Espectrometría de Masa (CEQUIBIEM), Argentina, in a Q Exactive HESI-Orbitrap coupled to a nano HPLC Easy-nLC 1000 (Thermo Scientific). MS/MS data were used to search the all the available *Trypanosoma cruzi* databases at Tritrypdb (version 30) [11].

### TcTASV-C- and EV- host cell interaction

Interaction assays were carried out by an ELISA-like assay, as described by Baida *et al* (2006) [61]. Briefly, macrophage (J774) or epithelial (Vero) cells were cultured overnight, washed with PBS-3% BSA and fixed with 1% paraformaldehyde in PBS for 15 minutes. The fixed cells were blocked with PBS-3% BSA-1% normal goat serum for 1 hour at room temperature and washed again. Recombinant proteins (TcTASV_GST_ or GST) were incubated for 1 hour at 37°C. The cells were washed and then incubated for 1 hour at 37°C with complete anti-TcTASV_GST_ sera, which recognizes both TcTASV_GST_ and GST proteins. Normal mouse serum was used as background control. Detection continued as for conventional ELISA technique. Three replicates per condition and three independent tests were carried out. Data were analyzed by Student t-test. A similar protocol was employed to assay the interaction EVs with Vero cells. Briefly, cells were incubated with freshly isolated EVs for 1.5 h at 37°C, washed and the interaction detected by a pool of sera developed against soluble and membrane antigens of trypomastigotes. Normal mouse sera and frozen EVs were used as controls.

### Interference of cell infection

The ability of rTcTASV-C to interfere with parasite infection on Vero cells was assessed *in vitro* by two different methods and with two *T*. *cruzi* strains. In both set ups cells were incubated with rTcTASV-C or GST (as a control), before infection. On one hand, 20000 Vero cells/well were incubated in p24 Wells (Costar) for 24 hs at 37°C. Then the cells were washed and incubated with recombinant proteins (TcTASV-C_GST_ or GST) in MEM 4% FBS at 37°C for 30 min. CL Brener trypomastigotes (10:1) were added to the cultures, and 18 h later uninternalized parasites were washed and infection proceeded for additional 48 hs. Cells were then fixed and stained with May-Grünwald Giemsa. At least 500 cells were counted in each technical replicate, and the presence of amastigotes registered. Data were normalized to infected (untreated) cells; 3 independent experiments were performed with 3 technical replicates each one. On the other hand, *T*. *cruzi* bloodstream trypomastigotes (Tulahuen strain) expressing *E*. *coli* β-galactosidase were used to infect treated Vero cells in p96 (Costar) in a relation of 10:1 [25,26]. After an overnight incubation (37°C, 5% CO2), cells were washed with PBS to remove non-infecting trypomastigotes and the culture maintained for additional 72 hs. Cells were then lysed with Igepal (1% v/v) and β-galactosidase activity was spetrophotometrically measured with the chromogenic substrate chlorophenol red β-D-galactopyranoside (CPRG). Reaction was read at 595 nm in a multi-plaque reader FilterMax F5 (Molecular Devices).

Purified *T*. *cruzi* bloodstream trypomastigotes (Tul- β-gal) were pretreated for 30 min at 37°C with anti-TcTASV-C sera (1/10) and then co-incubated (37°C, 5% CO2, 18 h) with Vero cells (ratio 10:1) in MEM–5% FBS in a 96-well plate format. Parasites pretreated with anti-GST or normal sera were used as controls. Cell culture and quantification of infection were the same as stated above. Untreated parasites were used to determine 100% of infection; 3 independent experiments were performed with 3 technical replicates each one.

### Plasmid DNA purification for immunization

For the preparation of plasmid DNA used in immunizations, *E*. *coli* DH5a containing a fragment of the TcTASV-C gene TcCLB.511675.3 (amino acids 233 to 305) cloned in pCI_Not_32 [12] or the plasmid VR1019 that contains the murine GM-CSF gene [26] were first grown as starter cultures in LB containing ampicillin at 37°C for 8 hours, then inoculated into a larger culture and grown O.N. and, finally, incubated additional 8 h in the presence of chloramphenicol (170 μg/ml) for amplification of plasmid copy number.

Plasmid DNA was purified with the QIAGEN EndoFree Plasmid Mega Kit (QIAGEN, GmbH, Germany) according to manufacturer’s instructions. Purified DNA was resuspended in TE endotoxin-free buffer and DNA concentration was estimated and stored at -20°C. For mice immunization, DNA was precipitated with ethanol and reconstituted at 1 μg/μl with sterile endotoxin-free PBS. The VR1019_GM-CSF plasmid was gently provided by Dr. Walter R. Weiss of the "Malaria Program and Pathology Division, Naval Medical Research Center," Maryland, United States.

### Mice immunization, challenge and parasitological follow up

C3H/He mice (n = 10 per group) were vaccinated with a prime (plasmid DNA) and boost (recombinant proteins) immunization protocol. Briefly, the first two doses consisted in intramuscular injections of plasmid DNA containing 100 μg of pCI_Not-TcTASV-C and 25 μg of VR1019_GM-CSF [12, 28]. The third and fourth doses consisted in subcutaneous injections of mixed TcTASV-C_GST_ and TcTASV-C_HIS_ (12.5 μg each one) with a colloidal suspension of aluminum hydroxide (Sigma). Control groups were immunized with 100 μg of the empty plasmid backbone pCI_Not_32 plus 25 μg of VR1019_GM-CSF (doses 1 and 2) and 25 μg of GST along with aluminum hydroxide (doses 3 and 4). Fifteen days after the last dose, 3 mice per group were sacrificed to evaluate cellular responses in spleen cells (cytokine production after culture) and the remainder 7 mice challenged with 100 bloodstream trypomastigotes of the RA strain by the intraperitoneal route [12]. Parasitemia was determined from day 7-on every 2–3 days until day 35. Mortality was daily monitored.

### Immune responses in vaccinated mice

Spleens of immunized mice were aseptically removed and homogenized. Red blood cells were lysed and cells cultured in RPMI 1640 supplemented with 2 mM L-glutamine, 100 U of penicillin/ml, 50 μg of streptomycin/ml, and 10% FCS at a concentration of 4 × 10^6^cells/ml in 24 well plates (Nunc). Cells were stimulated with TcTASV-C_GST_, GST (10 μg/ml), anti-CD3 (0.2 μg/ml) or solely maintained with culture medium (basal control) at 37°C in a humidified atmosphere of 5% CO_2_. After 72 h, supernatants were collected, and production of gamma interferon (IFN-γ) and interleukin-10 (IL-10) was evaluated by sandwich ELISA according to manufacturer's instructions (BD OptEIA, Pharmingen, San Diego, CA).

Serology against recombinant antigens or whole *T*. *cruzi* trypomastigote lysates was determined by ELISA, as we previously described [10,12]. Mice were bled by submandibular puncture to take serum samples 5 days before the immunization schedule start, 15 days after the last dose and at 42–62 days post infection. ELISA plates were sensitized with 50 ng of recombinant proteins or 100 ng of *T*. *cruzi* trypomastigote homogenates. Goat anti-mouse IgG, anti-IgG1 or anti-IgG2a conjugated to peroxidase (Thermo Fisher Scientific) were used as secondary antibodies. Reaction was revealed with 3,3’,5,5’-Tetramethylbenzidine (Sigma) and H_2_O_2_ in citrate buffer and read at 450 nm in a multi-plaque reader FilterMax F5 (Molecular Devices).

### Antibody-dependent complement mediated lysis of trypomastigotes

Parasites (500000/assay) were incubated with inactivated sera (53°C, 40 min) from vaccinated or control mice, for 1 h at 37°C, followed by treatment with fresh human sera (1:4) either with active or inactivated complement for an additional 1 h at 37°C [43]. Trypomastigote lysis was calculated by counting living, motile and unstained parasites in a Neubauer chamber after staining with Trypan blue.

### Mapping of B-cell epitopes

Putative B-cell epitopes in TcTASV-C proteins were predicted by Bepipred software [62]. The TcTASV-C epitopes identified in a previous work (peptide microarray screened with human antibodies) were also considered [29]. Peptides were purchased from Genscript and screened by ELISA. Briefly, plates were sensitized with 1 or 0.33 μg of peptide (for 96 or 384 plate format, respectively) in PBS O.N. Sera from vaccinated or infected mice were assayed at 1/100 dilution by triplicate. After incubation with a peroxidase- conjugated secondary antibody the reaction was developed as described above. The cut off was set up for each peptide as the media of the O.D. of the negative (uninfected unvaccinated) sera plus 3SD plus 10%. Reactivity of an experimental serum was classified positive for an X peptide, if the ratio between its O.D. for the peptide X and the cut-off value for peptide X, resulted in a value higher than 1 (i.e. O.D. sera for peptide X/ cut-off peptide X >1).

### Statistical analysis

For ELISA comparisons, we employed one-way ANOVA. Differences in the parasitemia between groups were determined by the Mann–Whitney U test. In survival analysis, groups were compared by the Log-rank test. In all cases, Graph Pad Prism version 5.01 (GraphPad Software, USA) was used and a P value below 0.05 was considered significant.

## Supporting information

S1 Dataset TcTASV-C protein sequences. TcTASV-C protein sequences retrieved from TriTrypDB and Genbank and used to feed WebLogo.
(TXT)

S1 Fig EVs from trypomastigotes were resolved by an Optiprep density gradient and analyzed by western blot. Gradient fractions were analyzed by Western blot using serum against TcTASV-C, HSP70 and TS (SAPA).
(TIF)

S2 Fig Proteomic analysis of EVs. (A) Genes identified in V2, V16 and both fractions. (B) Genes were grouped according to their putative localization: intracellular, surface or hypothetical proteins. (C) Surface and intracellular proteins presented according to their protein family or function.
(TIF)

S3 Fig TcTASV-C presents a dynamic pattern secretion on virulent strains. EVs purified from Y strain (TcII) trypomastigotes collected in consecutive days (Day 1, Day 2 and Day 3) from the same *in vitro* culture., were assayed for TcTASV-C expression. Tryp: trypomastigote; V2: large EVs; V16: small EVs; VF: vesicle-free fraction.
(TIF)

S4 Fig TcTASV-C secretion in bloodstream RA trypomastigotes. RA bloodstream trypomastigotes were isolated from mice in the parasitemia peak and purified by swimming. Equal volumes of blood from uninfected mice were processed in parallel, as control (Uninfected blood line). EVs were obtained as described for Fig 6. RA Cult Tryp 30: 30x10^6 in vitro cell-derived RA trypomastigotes; Tryp: trypomastigote; V2: large EVs; V16: small EVs; VF: vesicle-free fraction. CL-Brener Cult Tryp 30 : 30x10^6 in vitro cell-derived CL-Brener trypomastigotes; Uninfected blood: Normal mice blood processed like infected blood.
(TIF)

S5 Fig Trypomastigote-derived EVs interact with mammalian cells. **(A)** Reactivity against EVs of sera developed against *T*. *cruzi* membrane antigens (a-Tc membrane, left), anti-*T*. *cruzi* soluble proteins (a-Tc soluble, center) and both sera pooled (right) against total EVs derived from trypomastigotes. **(B)** Increasing amounts of EVs were incubated with to non-phagocytic professional cells (Vero cells). Binding was determined by an Elisa-like assay with the “pooled sera” shown in the right panel of A., followed by a colorimetric method. Values are means ± standard deviation of triplicates. **P < 0.01 compared with Frozen EVs values using Student’s t test.
(TIF)

S6 Fig Infected human sera reactivity against EVs from RA strain (TcVI). Western blot of RA EVs purified with differential centrifugation. Infected sera human were used to analyse the reactivity. Tryp: trypomastigote; V2: large EVs; V16: small EVs; VF: vesicle-free fraction.
(TIF)

S1 Table Reactivity of individual sera against TcTASV-C peptides. (XLSX)
