# Supplementary figures and images for: The protein family TcTASV-C is a novel Trypanosoma cruzi virulence factor secreted in extracellular vesicles by trypomastigotes and highly expressed in bloodstream forms
Source: PLoS Negl Trop Dis. 2018 May 4;12(5):e0006475. doi: 10.1371/journal.pntd.0006475 (PMC5955593; doi:10.1371/journal.pntd.0006475)

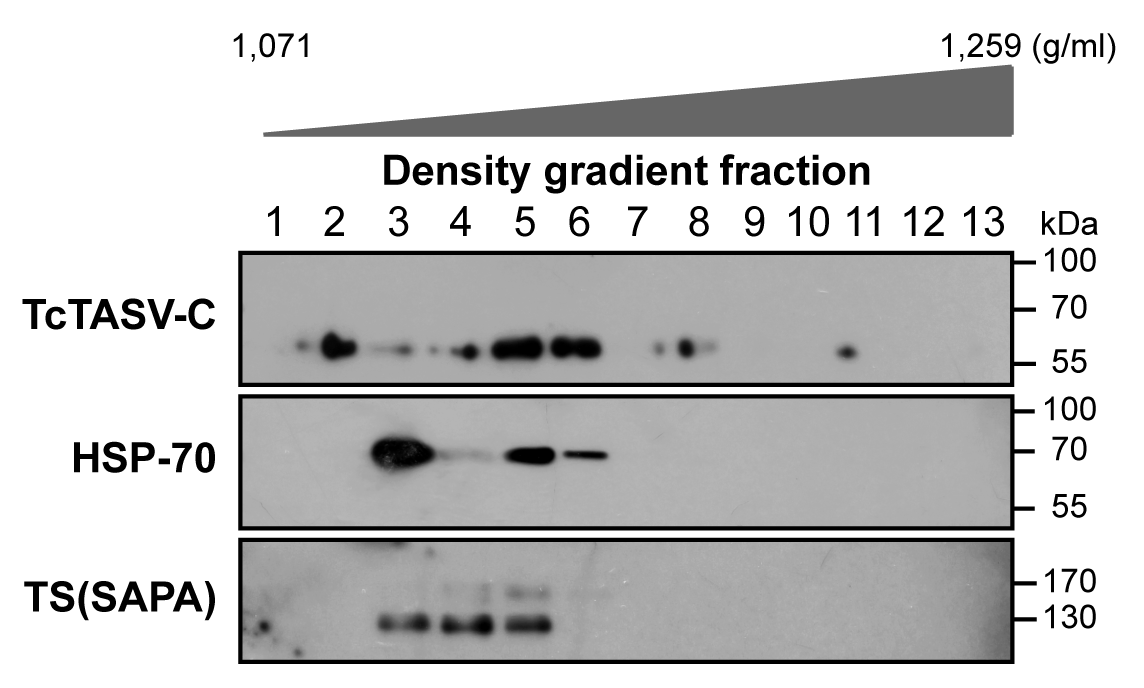

Supplement: S1 Fig — Gradient fractions were analyzed by Western blot using serum against TcTASV-C, HSP70 and TS (SAPA). (TIF) [file pntd.0006475.s002.tif]

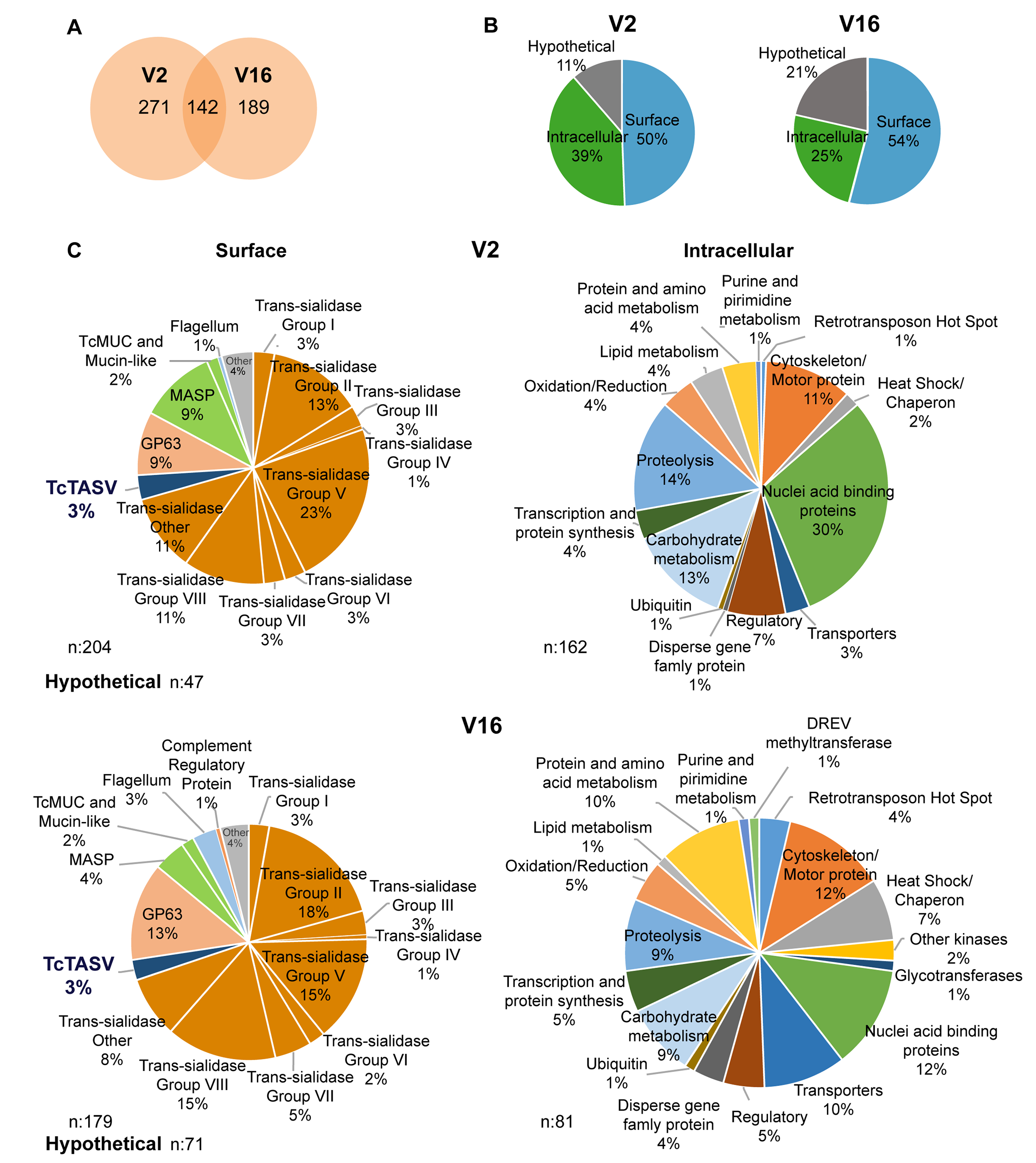

Supplement: S2 Fig — (A) Genes identified in V2, V16 and both fractions. (B) Genes were grouped according to their putative localization: intracellular, surface or hypothetical proteins. (C) Surface and intracellular proteins presented according to their protein family or function. (TIF) [file pntd.0006475.s003.tif]

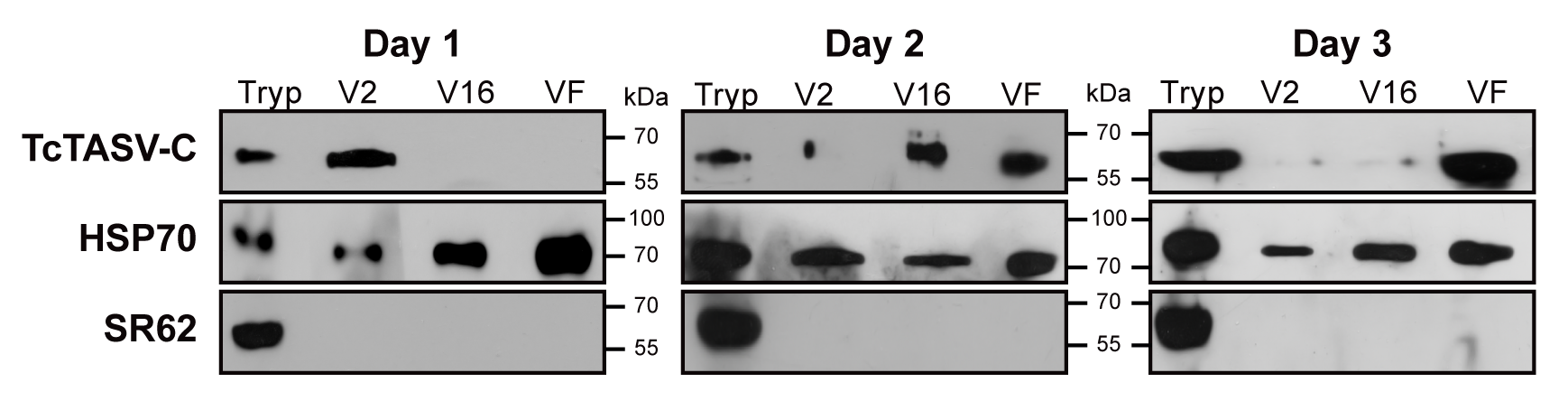

Supplement: S3 Fig — EVs purified from Y strain (TcII) trypomastigotes collected in consecutive days (Day 1, Day 2 and Day 3) from the same in vitro culture., were assayed for TcTASV-C expression. Tryp: trypomastigote; V2: large EVs; V16: small EVs; VF: vesicle-free fraction. (TIF) [file pntd.0006475.s004.tif]

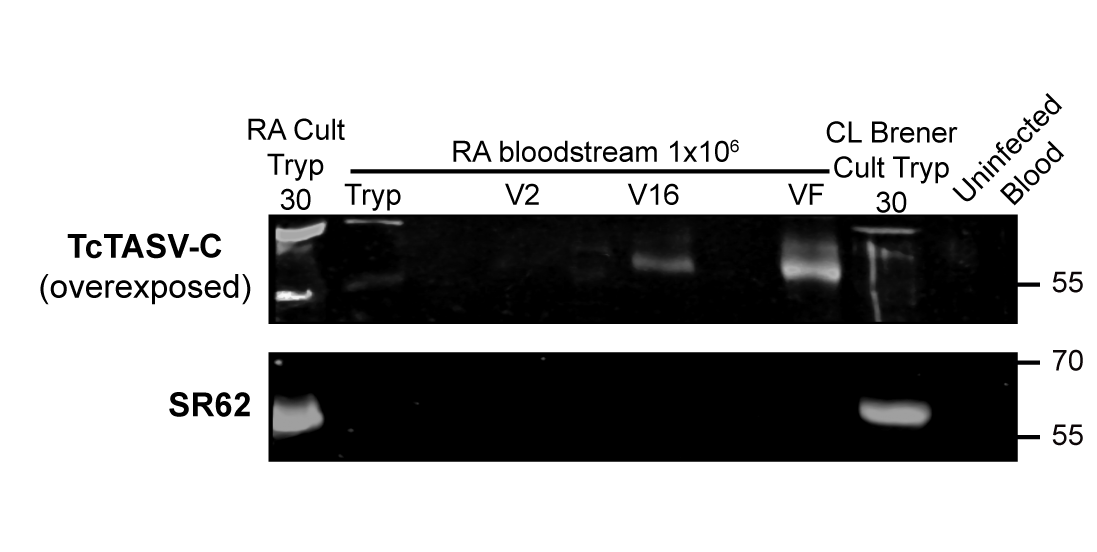

Supplement: S4 Fig — RA bloodstream trypomastigotes were isolated from mice in the parasitemia peak and purified by swimming. Equal volumes of blood from uninfected mice were processed in parallel, as control (Uninfected blood line). EVs were obtained as described for Fig 6. RA Cult Tryp 30: 30x10^6 in vitro cell-derived RA trypomastigotes; Tryp: trypomastigote; V2: large EVs; V16: small EVs; VF: vesicle-free fraction. CL-Brener Cult Tryp 30 : 30x10^6 in vitro cell-derived CL-Brener trypomastigotes; Uninfected blood: Normal mice blood processed like infected blood. (TIF) [file pntd.0006475.s005.tif]

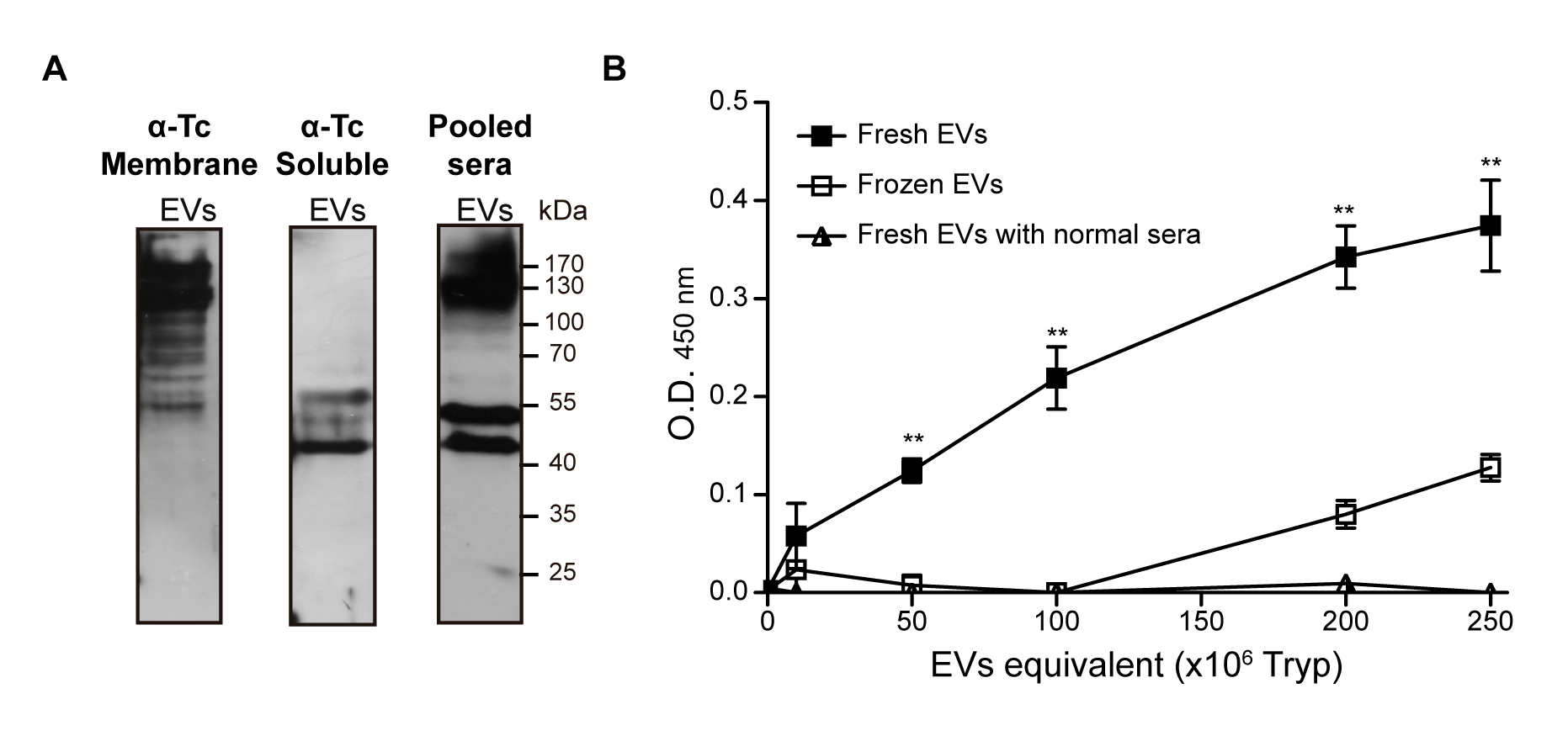

Supplement: S5 Fig — (A) Reactivity against EVs of sera developed against T. cruzi membrane antigens (a-Tc membrane, left), anti-T. cruzi soluble proteins (a-Tc soluble, center) and both sera pooled (right) against total EVs derived from trypomastigotes. (B) Increasing amounts of EVs were incubated with to non-phagocytic professional cells (Vero cells). Binding was determined by an Elisa-like assay with the “pooled sera” shown in the right panel of A., followed by a colorimetric method. Values are means ± standard deviation of triplicates. **P < 0.01 compared with Frozen EVs values using Student’s t test. (TIF) [file pntd.0006475.s006.tif]

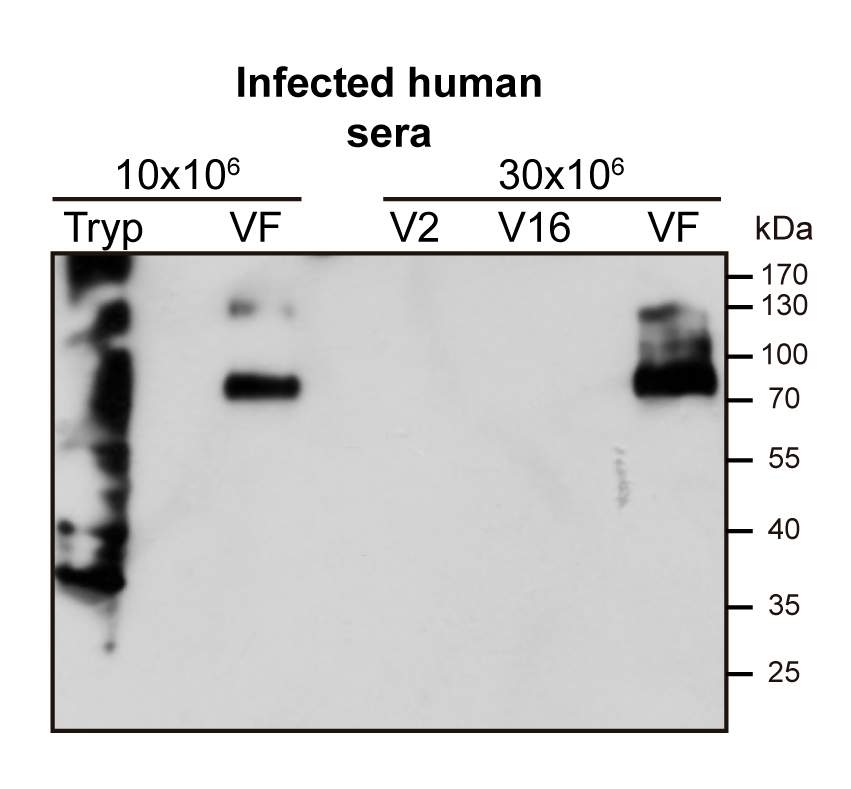

Supplement: S6 Fig — Western blot of RA EVs purified with differential centrifugation. Infected sera human were used to analyse the reactivity. Tryp: trypomastigote; V2: large EVs; V16: small EVs; VF: vesicle-free fraction. (TIF) [file pntd.0006475.s007.tif]
